# CellMapper: rapid and accurate inference of gene expression in difficult-to-isolate cell types

**DOI:** 10.1186/s13059-016-1062-5

**Published:** 2016-09-29

**Authors:** Bradlee D. Nelms, Levi Waldron, Luis A. Barrera, Andrew W. Weflen, Jeremy A. Goettel, Guoji Guo, Robert K. Montgomery, Marian R. Neutra, David T. Breault, Scott B. Snapper, Stuart H. Orkin, Martha L. Bulyk, Curtis Huttenhower, Wayne I. Lencer

**Affiliations:** 1 Division of Gastroenterology, Children’s Hospital and Harvard Medical School, Boston, MA 02115 USA; 2 Graduate Program in Biophysics, Harvard University, Cambridge, MA 02138 USA; 3 City University of New York School of Public Health, New York, NY 10027 USA; 4 Division of Genetics, Department of Medicine and Department of Pathology, Brigham and Women’s Hospital and Harvard Medical School, Boston, MA 02115 USA; 5 Center of Stem Cell and Regenerative Medicine, Zhejiang University School of Medicine, Zhejiang, 310058 People’s Republic of China; 6 Harvard Digestive Diseases Center, Harvard Medical School, Boston, MA 02115 USA; 7 Division of Endocrinology, Children’s Hospital and Harvard Medical School, Boston, MA 02115 USA; 8 Department of Gastroenterology, Brigham and Women’s Hospital, Boston, MA 02115 USA; 9 Division of Hematology/Oncology and Harvard Stem Cell Institute, Children’s Hospital and Harvard Medical School, Boston, MA 02115 USA; 10 Department of Pediatric Oncology, Dana-Farber Cancer Institute, Boston, MA 02115 USA; 11 Department of Biostatistics, Harvard School of Public Health, Boston, MA 02115 USA

**Keywords:** Cell type, Expression, Microarray, Genome-wide association study, Inflammatory bowel disease

## Abstract

We present a sensitive approach to predict genes expressed selectively in specific cell types, by searching publicly available expression data for genes with a similar expression profile to known cell-specific markers. Our method, CellMapper, strongly outperforms previous computational algorithms to predict cell type-specific expression, especially for rare and difficult-to-isolate cell types. Furthermore, CellMapper makes accurate predictions for human brain cell types that have never been isolated, and can be rapidly applied to diverse cell types from many tissues. We demonstrate a clinically relevant application to prioritize candidate genes in disease susceptibility loci identified by GWAS.

**Electronic supplementary material:**

The online version of this article (doi:10.1186/s13059-016-1062-5) contains supplementary material, which is available to authorized users.

## Background

Measuring gene expression in specific cellular subsets is key to understanding cellular function and differentiation and how these processes are disrupted during disease pathogenesis. However, there are steep technical challenges to obtaining pure populations of many cell types for expression profiling [1]. The human brain provides a clear example: many brain cell types display abnormal gene expression patterns when grown in culture [2] and must be acutely isolated from intact brain tissue to insure physiological relevance. Validated cell isolation protocols in mice often require the use of transgenic animals to label specific cell types [3–6] and are not applicable to humans. As a result, expression data are only available for a small fraction of the ~150 cell types [7] of the human central nervous system and this problem is similar for many other tissues.

One promising solution has been the development of computational methods to infer cell type-specific expression information directly from heterogeneous samples [8–19], such as undissociated tissue. These algorithms take advantage of the fact that the relative proportion of cell types varies from sample to sample, making it possible to statistically deconvolve expression changes in the underlying cell types. For many biological problems, it is not necessary to predict the total expression level of every gene in each cell type [8, 12–16], but rather the relative, or differential expression: specifically, which genes are strongly expressed in one cell type relative to others? It is these differentially expressed genes that frequently control cell differentiation, define cell-specific phenotypes, and provide the core signature of cell identity. By focusing on identifying differentially expressed genes, it turns a more complex model-fitting problem into a classification problem [9], opening the door to algorithms that may be more sensitive, especially for rare and difficult-to-isolate cell types. Several machine-learning algorithms have been developed to address this problem [17–19], each aimed at identifying genes with a similar expression profile to an established set of cell type-specific markers, referred to here as “query genes.” However, these algorithms all require very large training sets of both positive and negative control genes (≥10 of each) to define any cell type. This requirement poses a severe limitation for most biological applications, as it is difficult to curate such a large list of established marker genes for even well-studied cell types and impossible for many others.

Here, we present *CellMapper*, an algorithm optimized for sensitive identification of cell type-enriched genes using as little as a single query gene. We show that CellMapper can make accurate predictions for four human brain cell types that have never been isolated and cannot be addressed by any other computational method. We then apply our algorithm to a large compendium of 19,801 microarrays and identify genes specifically expressed in 30 diverse cell types of widespread importance in human biology, demonstrating that CellMapper can be readily used for cell types from many different tissues. Finally, we explore a clinically relevant application to prioritize candidate genes in loci identified by genome-wide association studies (GWAS). Our approach can be applied to any transcriptionally defined cell population using publicly available microarray data.

## Results and discussion

**Fig. 1:**
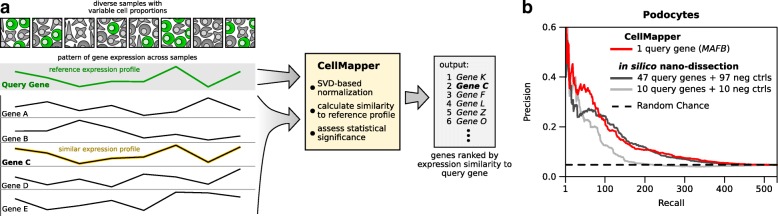
Overview and validation of CellMapper. **a** *Schematic* of the approach. CellMapper takes as input a cell type-specific query gene (*green*) and a set of gene expression data and finds genes with a similar expression profile to the query gene (e.g. “Gene C” above, *yellow profile*). **b** Performance comparison between CellMapper and the machine learning algorithm, in silico nano-dissection [17]. CellMapper and in silico nano-dissection were each applied to identify podocyte genes and evaluated based on the recovery of an experimentally defined set of podocyte genes [23]. In silico nano-dissection was applied using the training set selected by Ju et al. [17] for their analysis (46 query genes and 97 negative control genes) or a smaller training set of ten query genes and ten negative control genes (the smallest training set permitted by the algorithm, see “Methods”). CellMapper identified the experimentally defined podocyte genes with similar precision to in silico nano-dissection at all levels of recall, despite using only one query gene

CellMapper takes as input (1) a large set of gene expression data and (2) a query gene (or genes) specifically expressed in the cell type of interest and then estimates the probability that every other gene in the dataset is co-expressed with the query gene (Fig. 1a). Intuitively, CellMapper returns a gene list ranked according to the predicted expression level within the cell type of interest relative to others. The genes predicted to be most specifically expressed will be at the top of the rank list, followed by genes with decreasing levels of enrichment.

CellMapper is designed to make accurate predictions using as little as a single query gene, which can be selected from standard cell-specific markers employed by experimental techniques such as flow cytometry, immunohistochemistry, and promoter-driven conditional mouse knock out models. An important component of our algorithm is a filter based on singular value decomposition (SVD), which amplifies biologically informative signals in the expression data (Additional files 1 and 2). SVD-based filters have found diverse applications in biology, such as increasing sensitivity when reverse-engineering gene regulatory networks [20, 21] and controlling for population structure in GWAS [22], but have not been explored in the context of predicting cell type-specific expression before. In a test application to predict tissue-enriched genes (e.g. liver, heart, brain), we found that the CellMapper SVD filter both increased sensitivity and made the final algorithm consistently accurate across a range of tissues (Additional files 3 and 4). The SVD filter is likely beneficial for multiple reasons (discussed further in Additional file 1), such as enhancing subtle biological signals, reducing batch effects, and increasing robustness to bias in dataset sample composition (Additional file 5).

As a first test of CellMapper’s performance, we compared it to in silico nano-dissection [17]—the most recent and sensitive machine-learning algorithm to predict cell type-enriched genes from heterogeneous microarray data. In silico nano-dissection was previously shown to have good prediction accuracy for kidney podocytes using a large set of human kidney microarray data [17] and so we applied CellMapper to this same dataset using the query gene *MAFB*. We found that CellMapper identified experimentally-defined podocyte genes [23] with similar precision to in silico nano-dissection at all levels of recall (Fig. 1b), despite using a much smaller training set of query genes (1 query gene for CellMapper versus 47 query genes plus 97 negative control genes for in silico nano-dissection). This finding was consistent when CellMapper was run using podocyte marker genes other than *MAFB* as the query gene (Additional file 6). We then repeated in silico nano-dissection with a smaller training set of ten query genes and ten negative control genes (the smallest training set permitted by the algorithm). When using this smaller training set, we observed a decrease in performance for in silico nano-dissection, such that it performed noticeably worse than CellMapper (Fig. 1b, light gray line). Thus, CellMapper achieved similar accuracy to in silico nano-dissection while requiring substantially fewer query genes.

### CellMapper is accurate for cell types that cannot be approached by other methods

We next applied CellMapper to identify genes expressed in four cell types of the central nervous system—GABAergic neurons, noradrenergic neurons, serotonergic neurons, and NG2 glia—using human microarray data from the Allen Brain Atlas [24]. These cell types were selected because they are relevant to human disease [25, 26], but have not been isolated from adult humans for expression analysis before. In addition, the relatively limited knowledge of specific markers for these cell types makes it difficult to apply algorithms that require a large training set, such as in silico nano-dissection. The Brain Atlas provides a unique opportunity to fill this gap in expression data using CellMapper: it includes microarrays from 900 distinct sub-regions of the adult human brain, each with varying cellular composition, and it contains sufficient signal to differentiate genes expressed in the major brain cell classes (neurons, astrocytes, oligodendrocytes, and microglia) [24] and likely other brain cell types. We applied CellMapper to search the Brain Atlas dataset using query genes specific to GABAergic neurons (*GAD1*), noradrenergic neurons (*SLC6A2*), serotonergic neurons (*SLC6A4*), and NG2 glia (*PDGFRA*). Each of these genes are standard markers for their respective cell type, and three have been previously used to experimentally isolate the cell type for expression profiling in mice [3, 5, 6]. This analysis returned between 61 and 211 genes per cell type at a false discovery rate (FDR) of 0.01 (Additional file 7).

**Fig. 2:**
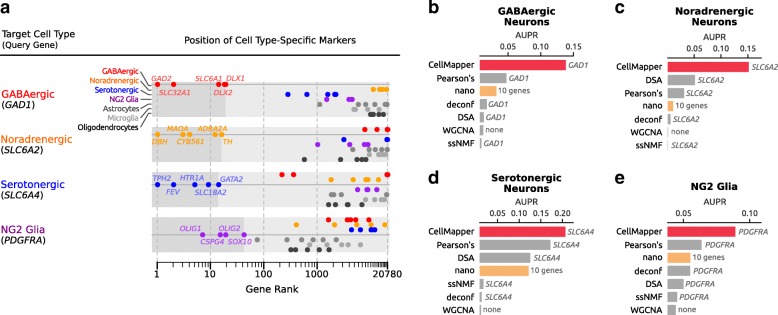
Application of CellMapper to brain cell types that are difficult to address by other methods. **a** CellMapper was applied to the Allen Brain Atlas dataset using the indicated query genes for four brain cell types. *Dot charts* display the rank of literature-defined cell-specific markers (positive controls) within CellMapper’s predictions for each cell type. *Dots* are colored based on their known primary cell type of expression. *Dark gray shading* covers the area (rank list) required to identify all positive control genes for each cell type. A similar analysis using query genes other than *GAD1*, *SLC6A2*, *SLC6A4*, and *PDGFRA* for the four cell types is provided in Additional file 16. **b**–**e** Performance evaluation of CellMapper and other computational methods to recover genes expressed in the four brain cell types. Each method was evaluated based on the recovery of an experimentally-defined [3–6] set of cell type-enriched genes in mouse, as quantified by the area under the precision recall curve (AUPR). WGCNA returns several modules of gene co-expression, the best performing WGCNA module is plotted for each cell type

To evaluate the accuracy of our results, we took two complementary approaches. In the first, we examined CellMapper predictions for literature-defined markers (positive controls) of each cell type, including GABAergic neurons (*GAD2*, *SLC6A1*, *SLC32A1*, *DLX1*, and *DLX2*), noradrenergic neurons (*DBH*, *TH*, *MAOA*, *CYB561*, and *ADRA2A*), serotonergic neurons (*FEV*, *TPH2*, *HTR1A*, *SLC18A2*, and *GATA2*), and NG2 glia (*CSPG4*, *OLIG1*, *OLIG2*, and *SOX10*). CellMapper correctly associated all positive control genes with the expected cell type (Fig. 2a), while excluding markers of the other cell types. In addition, CellMapper excluded genes known to be absent in these cell types, such as markers for astrocytes (*S100B*, *GFAP*, *SLC1A3*, *FGFR3*, *AQP4*, and *GLUL*), microglia (*CX3CR1*, *AIF1*, *CSF1R*, *FCGR1A*, and *TREM2*), and mature oligodendrocytes (*PLP1*, *MOBP*, *MBP*, *MAG*, and *CMTM5*). In the second approach, we asked whether CellMapper predictions for each cell type were enriched for genes associated with these cell types as measured by expression profiling in mice [3–6], where these cells have been experimentally isolated. We found that our predictions for GABAergic neurons, noradrenergic neurons, serotonergic neurons, and NG2 glia were each significantly enriched for genes expressed by the corresponding cell type in mice (*p* = 8 × 10^−24^, *p* = 3 × 10^−9^, *p* = 7 × 10^−32^, and *p* = 5 × 10^−15^, respectively; Fisher’s exact test), and these findings were consistent when CellMapper was re-applied using truncated versions of the Allen Brain Atlas dataset (Additional file 8, left) or an alternative brain microarray expression compendium (Additional file 8, right).

We next attempted to apply a range of existing computational methods to this problem, including in silico nano-dissection [17], weighted gene co-expression network analysis (WGCNA) [10], and three “computational deconvolution” algorithms from the *CellMix* [12] R package: deconf [15], the digital sorting algorithm (DSA) [13], and semi-supervised non-negative matrix factorization (ssNMF) [14]. Of these, only in silico nano-dissection was designed to predict genes expressed selectively in difficult-to-isolate cell types (similar to CellMapper); all other algorithms can be used for this purpose, but were motivated by distinct biological problems and are not expected to perform optimally in this application (Additional file 9). We applied each algorithm to the Brain Atlas dataset using the same query genes as above, except for in silico nano-dissection, which required at least ten genes, and WGCNA, which is unsupervised and does not accept query genes. Then we assessed how accurately each algorithm identified the experimentally-defined cell type genes in mice [3–6], as quantified by the area under the precision-recall curve (AUPR). CellMapper consistently outperformed all other algorithms (Fig. 2b–e), with the other algorithms showing particularly poor performance for GABAergic neurons and NG2 glia (Fig. 2b, e). Supporting this conclusion, the other algorithms were also unable to identify standard cell type markers for most of these cell types (Additional file 10). One explanation for this difficulty is that these four cell types are relatively uncommon—comprising less than 10 % of total cells in most regions of the brain—and thus pose a particularly challenging problem for computational prediction. For comparison, all algorithms performed reasonably well for the major brain cell classes (neurons, astrocytes, oligodendrocytes, and microglia), with CellMapper and in silico nano-dissection consistently outperforming the others (Additional file 11). Thus, CellMapper can make accurate predictions for rare cell types that cannot be addressed by other methods.

### Application to diverse cell types

We also tested CellMapper on a large panel of additional cell types (Additional file 12), this time extending our analysis to include non-brain cell types, with multiple representatives of all major cell classes (neural, epithelial, connective tissue, muscle, and hematopoietic). In order to apply CellMapper to cell types outside the brain, we gathered three additional large microarray datasets. The first two are meta-analyses of gene expression in human [27, 28], each of which integrated expression data from a wide range of sample types—including whole organs, purified cell populations, and cell lines. The third is a meta-analysis of gene expression in mouse [29] and includes microarrays from a similarly diverse set of samples. Combined, these additional datasets comprise 16,090 microarray samples and contain expression data for 20,411 genes. This large microarray compendium covers essentially every mammalian tissue and contains samples of most cell types in purified and/or mixed form.

**Fig. 3:**
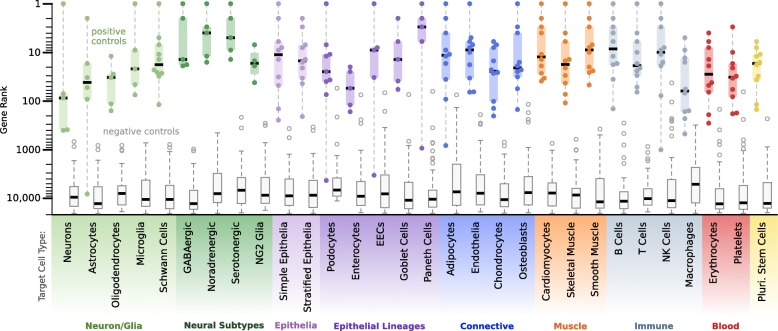
CellMapper is accurate across diverse cell types. CellMapper was applied using query genes for 30 cell types (Additional file 12); *Tukey boxplots* display the rank of 4–10 literature curated markers (positive controls; Additional file 13) and ≥48 negative control genes (Additional file 13 and housekeeping genes from [30]) for each cell type, demonstrating that CellMapper sensitively identified established cell type markers in every case. Filled circles represent the rank of all positive control genes; *open gray circles* represent negative control genes that fall outside 1.5 times the interquartile range of the other negative control genes (“outliers”). In only eight instances (0.5 %) was a negative control gene identified within the top 100 predictions for a cell type. *EECs* enteroendocrine cells

We curated one query gene for each cell type and applied CellMapper to search the microarray datasets using these query genes (Additional file 12). This analysis resulted in a mean of 331 cell type-enriched genes predicted per cell type (FDR ≤ 0.01; Additional file 1). Again, the quality of our results was evaluated using literature-curated positive control genes (both the positive control genes and references used to select them are described in Additional file 13) as well as a set of negative control genes, which included cell-specific markers for non-target cell types (Additional file 13, bold genes) and a reference set of housekeeping genes [30]. For every cell type, CellMapper identified over half of the positive control genes within the top 100 predictions (Fig. 3), and excluded almost every negative control gene. In total, 205 out of 236 positive controls were ranked within the top 100 predictions for the correct cell type (86.9 %) and all but six were ranked within the top 500 predictions (97.5 %). Thus, CellMapper is accurate for both single-organ and multi-organ cell types and for cell types difficult to isolate or culture (e.g. Schwann cells, Paneth cells). For applications of CellMapper to additional cell types, both the algorithm and pre-processed microarray data are available as an R package in Bioconductor.

### Prioritizing candidate genes affecting human disease

GWAS have linked numerous human genetic variants, such as single nucleotide polymorphisms (SNPs), to different traits and diseases. Although each associated variant implicates a genomic region that can include as many as ten or more genes, only one is typically relevant to disease pathogenesis [31]. One successful approach to prioritize GWAS candidate genes has been to look for genes that are selectively expressed in the tissue(s) or cell type(s) most relevant to disease pathogenesis [32, 33]. CellMapper offers several advantages for this method of analysis because it can profile almost any relevant cell type, as long as one marker gene is known.

**Fig. 4:**
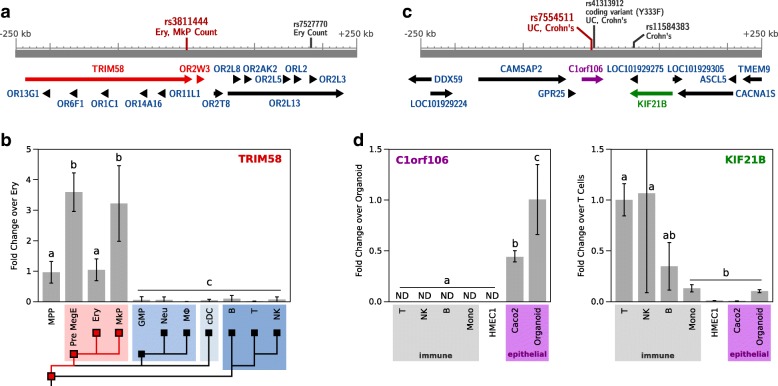
Using CellMapper to prioritize GWAS disease genes. **a** The genetic locus surrounding sentinel SNP rs381144, associated with erythrocyte (Ery) and platelet (MkP) cell number. Other relevant SNPs in the region are shown. All genes predicted for expression in erythrocytes and platelets are displayed in *red*. **b** *TRIM58* expression in primary mouse hematopoietic cells by qRT-PCR. *MPP* multi-potent progenitor, *PreMegE* pre-megakaryocyte-erythrocyte, *Ery* erythrocyte, *MkP* megakaryocyte/platelet, *GMP* granulocyte-monocyte progenitor, *Neu* neutrophil, *MΦ* macrophage, *cDC* conventional dendritic cell, *B* B cell, *T* T cell, *NK* natural killer cell. **c** The genetic locus surrounding sentinel SNP rs7554522, associated with inflammatory bowel disease (IBD). Genes colored in *purple* are predicted for simple epithelial cells, genes colored in *green* are predicted for T and NK cells. **d** *C1orf106* and *KIF21B* expression in human primary cells and cell lines. *Mono* monocyte, *HMEC1* endothelial cell line, *Caco2* colon epithelial cell line, *Organoid* primary epithelial organoid from small intestine biopsy. All bars are mean +/− SD (n = 3–7 independent biological replicates) and letters indicate statistically significant differences between groups (*p* ≤0.05, Tukey’s honest significant difference test)

As a proof of principal, we applied CellMapper to prioritize genes from two recent GWAS meta-analyses of erythrocyte [34] and platelet [35] phenotypes, two examples where high quality GWAS data are available and the relevant cell type is unambiguous. CellMapper predictions for erythrocytes and platelets were more than tenfold enriched within 10 kb of SNPs associated with red blood cell and platelet phenotypes, respectively (*p* = 1.0 × 10^−9^, *p* = 6.9 × 10^−5^; Fisher’s exact test), providing initial evidence that CellMapper might be used to highlight genes from these studies. Among the GWAS loci for erythrocyte and platelet genes, we found 30 candidates predicted to be selectively expressed in the relevant cell type (Additional file 14). One gene that stood out was *TRIM58* because it is in a locus associated with both erythrocyte and platelet cell number (Fig. 4a) and predicted to be selectively expressed in both cell types with high confidence (FDR < 10^−15^). To test our expression prediction, we measured *TRIM58* expression across hematopoietic cells by quantitative real-time polymerase chain reaction (qRT-PCR), and found that it was expressed exclusively in eryothrocytes, platelets, and their common progenitors (Fig. 4b). This result implicates a role for TRIM58 in the developmental program for erythrocytes, as just recently described [36], and for platelets.

We next applied CellMapper to analyze GWAS results for the chronic inflammatory bowel diseases (IBD), a complex set of diseases involving many cell types, including some that lack gene expression profiles. We focused on the 163 IBD susceptibility loci identified by Jostins, et al. [37], 38 of which lack any candidate gene(s) highlighted by previous prioritization strategies. Genes predicted by CellMapper to be differentially expressed in T cells, B cells, NK cells, and platelets were more than fivefold enriched among genes located within 10 kb of IBD SNPs (*p* < 0.01 for all cell types), highlighting the well-known relevance of the three lymphocyte cell types to IBD [38] and supporting the view that platelets also play an active role in disease pathogenesis [39]. We searched IBD loci for genes predicted to be differentially expressed in these four cell types and four others that contribute to IBD [38]—macrophages, simple epithelial cells, goblet cells, and Paneth cells. This analysis highlighted 64 novel candidates and provided additional support for 75 previously implicated genes (Additional file 14). Example candidates highlighted by CellMapper are *C1orf106* and *KIF21B* (Fig. 4c), two genes in the same locus predicted to be enriched in simple epithelial cells and in T and NK cells, respectively. As before, we verified our expression predictions by qRT-PCR, this time using human immune cell types isolated by FACS, cultured endothelial and epithelial cell lines, and primary intestinal epithelial organoids (Fig. 4d). The results confirm epithelial expression of *C1orf106*, and T and NK cell expression of *KIF21B*. This example illustrates another benefit of CellMapper as a prioritization strategy for GWAS: CellMapper can be used to not only prioritize candidate genes, but also to suggest which cell type(s) might be affected for each candidate. *C1orf106*, the gene we discovered to be epithelia-specific, is particularly interesting as an IBD candidate because rare coding variants in this gene have been associated with an increased risk for IBD [40].

To assess whether CellMapper could also be used to prioritize candidates for other diseases, we comprehensively searched for enrichment of disease candidate genes among our top predictions for each of the 30 cell types. We considered both genes linked to human genetic disorders in Online Mendelian Inheritance in Man [41] (OMIM) and genes in disease susceptibility loci identified by GWAS [42]. Both OMIM genes and GWAS candidates were significantly enriched in the top 200 predictions across all cell types (*p* = 1.8 × 10^−20^ and 4.3 × 10^−19^, respectively; Fisher’s exact test). Furthermore, we frequently found that genes linked to individual diseases were enriched in the top predictions for specific cell types (Additional file 15) and these disease-cell type associations primarily highlighted cell types with an established role in disease pathology. These results demonstrate the potential of CellMapper to prioritize genes for many other human diseases.

## Conclusions

We developed CellMapper as an approach to obtain the gene expression profiles unique to individual cell types. Such data are often required for continued advances in biology and medicine. Unlike experimental methods to define cell type-specific gene expression, CellMapper can be rapidly applied using existing publicly available microarray data and knowledge of only a single cell-specific marker gene. Markers can be used to delineate not only individual cell lineages (*DEF5A*+ Paneth cells), but also larger classes of cells with similar function (*KRT8*+ simple epithelia), thus allowing the level of resolution to be tailored to the needs of each specific biological question. Furthermore, CellMapper is effective for cell types that have never been isolated before, providing an opportunity to fill gaps in available expression data.

Our results establish CellMapper as a general and accurate method, and a resource for diverse applications in biology and medicine. Not only can CellMapper identify new cell type-specific markers, but the complete set of genes predicted to be enriched in a cell type can be used for many applications, such as inferring transcription factor binding motifs [43] or identifying biological pathways particularly active in a given cell type. There is also value in combining cell type-specific expression with other sources of high throughput data in order to suggest novel gene candidates for a pathway. We show this application by integrating cell type expression with GWAS data, but a similar approach could be applied to other problems, such as to identify genes of a particular class or function (e.g. membrane trafficking genes) that are strongly expressed by a specific cell type (e.g. polarized epithelial cells).

We found that CellMapper outperformed other computational methods and provided accurate predictions for difficult-to-isolate cell types where the other methods failed. This result highlights the need to develop computational tools optimized for the specific questions being asked. For example, the three “computational deconvolution” algorithms we tested (DSA, deconf, and ssNMF) were originally created to address problems distinct from CellMapper: in diseases where the proportion of different cell types varies according to disease state (e.g. cancer, Huntington’s disease), these methods can distinguish between changes in gene expression caused by changes in cell type frequency from those caused by altered gene expression within the individual cell types. This question is biologically important and clinically relevant, but cannot be addressed by CellMapper. Similarly, many algorithms have been created to predict genes in a co-regulated biological pathway based on co-expression analysis. CellMapper could be applied to identify genes in a similar biological pathway as a query gene, but we would not expect it to compete favorably with existing algorithms [44, 45] designed for this purpose. For the important question of identifying which genes are most selectively expressed in a cell type, however, CellMapper excels.

A built-in limitation of CellMapper, and related approaches, is that they depend on the availability of cell-specific marker genes and large, representative expression datasets. Fortunately, marker genes have been established for a wide variety of cell types and the requirement of a single marker gene is no greater than that needed by experimental approaches such as by FACS and immunohistochemistry. The availability of expression data will be most limiting for rare cell types that populate a single organ, but we showed that CellMapper can still separate genes expressed in closely related cell types such as neuron subtypes and intestinal epithelial lineages. Another limitation is that CellMapper has currently only been validated for use with microarray data. Certain classes of genes, such as long non-coding RNAs, are not well represented in most microarray platforms. Many algorithms that explore gene co-expression relationships have translated well to RNA sequencing (RNA-Seq) data [46], and CellMapper in principle could be adapted for RNA-Seq to allow for more complete coverage of the transcriptome.

## Methods

### Dataset acquisition and processing

Four large microarray datasets were gathered for this study, each comprising numerous microarray experiments performed on a single Affymetrix platforrm. Two of the datasets were downloaded from ArrayExpress (accession numbers E-MTAB-62 and E-MTAB-27); these contain 5372 experiments on the Human Genome U133A array [28] and 1323 on the Mouse Genome U74A array [29]. The third dataset was kindly provided by J. Engreitz, and contains 9395 experiments on the Human Genome U133 Plus 2.0 array [27] (now available on GEO: GSE64985). RMA-normalized expression values were adjusted to reduce the influence of technical bias (i.e. variation in hybridization conditions or starting material) using the R package bias 0.0.3 [47]. In addition, a fourth normalized dataset was downloaded from the Allen Brain Atlas [24] and analyzed without further processing. To generate an intestine-specific subset of microarray data (used for the four intestinal epithelial lineages), all samples from the Engreitz et al. [27] dataset with the terms COLON* or INTESTIN* in the title or abstract of the GEO submission were included, as well as samples from the Lukk et al. [28] dataset that were annotated by the authors as from “colon,” “colon mucosa,” or “small intestine.” Kidney podocytes were analyzed using the same datasets as in Ju et al. [17] (GEO accessions: GSE32691, GSE35488, GSE37455, GSE37460, and GSE47185).

Probesets were mapped to Entrez gene identifiers with the Bioconductor annotation packages hgu133a.db [48] and mgu74av2.db [49], and values for probesets mapping to the same gene were averaged to produce a single expression measurement for each gene. Mouse Entrez gene identifiers were then mapped to the corresponding human orthologs using a hierarchy of orthology predictions: first, mouse genes were mapped to human orthologs using orthology predictions from the Mouse Genome Institute (MGI); second, genes not mapped by MGI were then matched to human genes with an identical HGNC name; third, the remaining genes were mapped using orthology predictions from Inparanoid, then Ensembl, and finally Homologene. This hierarchical mapping strategy ensured reasonable specificity while maintaining greater sensitivity by using multiple orthology databases. All orthology predictions were downloaded from the HGNC Comparison of Orthology Predictions (HCOP) database [50].

### Performance evaluation of computational algorithms

This section describes all performance evaluation to compare between computational algorithms in the main text. Each algorithm was tested against a gold standard of experimentally defined cell type-enriched genes in mice. Podocyte gold standards were from Table S1 of Brunskill et al. [23]. Serotonergic gold standards were from Table 1 of Dougherty et al. [5]. GABAergic neuron gold standards were all genes with a mean expression at least threefold higher in the *GAD1*+ samples from Sugino et al. [3] than in other samples. Gold standards for NG2 glia and the major brain cell class were all genes with a mean expression at least threefold higher in the purified cell type than in the other samples from Zhang et al. [6]. Noradrenergic gold standards were all genes from Table S2 of Grimm et al. [4] with a “Ratio LC” greater than ten (more than tenfold higher expression in Noradrenergic neurons than the whole-brain reference) and a “Ratio LC” at least fivefold greater than the ratio for other neuron subtypes. Gold standard genes from mouse were then mapped to the orthologous human genes using the procedure described in the “Dataset Acquisition and Processing” section, above.

To predict cell type-enriched genes with in silico nano-dissection: in silico nano-dissection was applied using the nano-dissection web server (nano.princeton.edu) and either the “Renal Microdissections” or “Allen Brain Atlas” datasets. For podocytes, we used the positive and negative control training sets from the original nano-dissection paper (47 positive and 97 negative control genes) or a smaller training set of ten positive and ten negative control genes, which included the ten podocyte markers listed in Additional file 13 plus *MAFB* as positive controls and markers for the other major kidney cell types as negative controls (negative controls: *CDH5*, *KDR*, and *TEK* for endothelia; *ACTA2*, *CD34*, and *PDGFRB* for mesangial cells; *AQP1*, *SLC12A1*, *SLC12A3*, and *UMOD* for tubule cells). Positive controls for the brain cell types were: GABAergic neurons (*GAD1*, *GAD2*, *SLC32A1*, *SLC6A1*, *DLX1*, *DLX2*, *ABAT*, *ARX*, *GABBR2*, and *NPY*), noradrenergic neurons (*SLC6A2*, *DBH*, *MAOA*, *CYB561*, *TH*, *ADRA2A*, *SLC18A2*, *SLC31A1*, *TFAP2A*, and *TFAP2B*), serotonergic neurons (*SLC6A4*, *SLC18A2*, *FEV*, *TPH2*, *HTR1A*, *GATA2*, *GATA3*, *TPH1*, *HTR1B*, and *DDC*), NG2 glia (*PDGFRA*, *CSPG4*, *SOX10*, *OLIG1*, *OLIG2*, *SOX8*, *SOX3*, *GPR17*, *C1QL2*, and *NKX2-2*), neurons (*L1CAM*, *SYT1*, *NRXN1*, *SNAP25*, *SLC12A5*, *TUBB3*, *ENO2*, *STMN2*, *SYN2*, and *SYN1*), astrocytes (*ALDH1L1*, *FGFR3*, *GFAP*, *GJB6*, *F3*, *SLC1A3*, *AQP4*, *SLC1A2*, *GLUL*, and *GJA1*), oligodendrocytes (*MOG*, *MOBP*, *PLP1*, *GJC2*, *MAG*, *MAL*, *OLIG2*, *SOX10*, *MBP*, and *CNP*), and microglia (*PTPRC*, *CX3CR1*, *CD68*, *CSF1R*, *AIF1*, *P2RY13*, *FCGR1A*, *FCGR2B*, *SLC2A5*, and *TREM2*). In the in silico nano-dissection paper, the major brain cell types were analyzed using training sets curated by the Human Protein Reference Database (HPRD); we found that these HPRD training sets resulted in extremely low AUPRs, which is why we curated custom markers to apply nano-dissection to the major brain cell types. As negative controls for the four major brain cell classes, we used all markers for the other three brain cell classes. As negative controls for the four neural subtypes, we included markers for the other three subtypes as well as a set of genes expressed in non-target brain glia (*ALDH1L1*, *SLC1A2*, *SLC1A3*, *GFAP*, *GJB6*, *FGFR3*, *AQP4*, *GJA1*, *GLUL*, *F3*, *PTPRC*, *CX3CR1*, *AIF1*, *CSF1R*, *FCGR1A*, *TREM2*, *FCGR1B*, *P2RY13*, *SLC2A5*, *CD68*, *MOG*, *PLP1*, *MOBP*, *SOX10*, *MAG*, *MBP*, *GJC2*, *OLIG2*, *CNP*, and *MAL*).

To predict cell type-enriched genes with the digital sorting algorithm (DSA), deconf, or semi-supervised non-negative matrix factorization (ssNMF): DSA, deconf, and ssNMF were applied to the Brain Atlas data using the wrappers provided in the CellMix [12] R package. There are two distinct options in the CellMix package for ssNMF: ssKL and ssFrobenius. All reported AUPRs are for the results obtained with ssFrobenius as this method resulted in a consistently higher AUPR than ssKL.

To predict cell type-enriched genes with WGCNA: WGCNA has been previously applied to the Allen Brain Atlas dataset [24], and we gathered the 13 modules identified by this previous analysis (Table S4 of [24]). AUPR was calculated for each individual module after ranking all genes according to their module membership (the correlation between each gene and the module eigengene) and then the maximum AUPR achieved by any module was reported.

### The CellMapper algorithm

Below is a description of the CellMapper algorithm; a more detailed discussion and rationale for the CellMapper SVD filter is provided in Additional file 1.

#### Singular value decomposition (SVD) filter

Expression data (m genes × n samples) were scaled such that each gene had a mean expression of 0 and standard deviation of 1. The scaled expression matrix, *X*, was then factored by SVD:

$$ {X}_{m\times n}\kern0.5em =\kern0.5em {U}_{m\times n}{E}_{n\times n}\kern0.5em {V}_{n\times n}^T $$

where *U* and *V* contain the right- and left-singular vectors of *X* and *Σ* contains the singular values of *X* in decreasing order along the diagonal. These SVDs were then used to weight results using two components. First, singular values are scaled by an exponent, α, in order to reduce the relative importance of the early singular vectors. Alpha can fall between 1 (no scaling) and 0 (all singular values have equal weight). We investigated choices of α (Additional file 4) and selected α = 0.5 for all analyses described in this paper. Second, the singular values are multiplied by a weight term that smoothly filters out singular vectors where the query genes are not well separated from the rest of the genome:

$$ \begin{array}{c}\hfill {\sigma}_k\hbox{'}={\sigma_k}^{\alpha}\times \left|{w}_k\right|\hfill \\ {}\hfill {w}_k={\displaystyle \sum_{g\in (querygenes)}} tanh\left({u}_k^g\right)\hfill \end{array} $$

where σ_k_ represents singular value k, α is the singular value scaling factor, and *u*_*k*_^*g*^ is the loading of gene g in singular vector k, normalized so that the mean *u*_*k*_ is 0 with a standard deviation of 1. The rationale for our SVD filter, and the selection of the parameter α, are described in detail in Additional file 1. After filtering the singular values, the data were transformed back:

$$ {X}_{m\times n}={U}_{m\times L}{\varSigma}_{L\times L}\hbox{'}{V}_{n\times L}^T $$

where *Σ'* is the transformed singular value matrix, and L is the number of singular vectors to keep during the filter (L ≤ n). We selected L to trim singular vectors that account for less variance than an individual sample in the original dataset (Kaiser’s criterion), thereby removing singular vectors that mainly account for noise.

#### Calculate similarity to reference expression profile

After the SVD filter is applied, we calculate the mean of the Fisher-transformed correlation of each gene, g, with all query genes:

$$ {\overline{z}}_g=\frac{1}{2N}{\displaystyle \sum_{Q\in querygenes}} ln\left(\frac{1+{\rho}_{gQ}}{1-{\rho}_{gQ}}\right) $$

where *ρ*_*gQ*_ is the Pearson’s correlation of gene g with query gene Q and N is the total number of query genes.

#### Assessing statistical significance

We first standardize the Fisher-transformed correlations by their median and median absolute deviation (MAD):

$$ {S}_g=\frac{{\overline{z}}_g- median(z)}{1.4826\times MAD(z)} $$

*P* values are then calculated for S_g_ using the standard normal distribution; this produces equivalent results to a permutation test, as S_g_ closely approximates a standard normal distribution when sample labels are scrambled (R^2^ = 0.999996 in a normal QQ plot). The SVD filter, query-driven search, and statistical significance are calculated separately for each microarray platform, then *p* values from all three platforms for each gene are pooled together using Stouffer’s Z-score method.

### Prioritizing GWAS candidates with CellMapper

We prioritized candidate genes located near GWAS SNPs in two phases. In the initial phase, we determined which cell types are “priority” cell types for a particular GWAS disease. We first searched for GWAS positional candidates enriched in the top 200 cell type-enriched genes from each CellMap cell type (*p* ≤ 0.05; Fisher’s exact test adjusted for multiple hypothesis testing with Holm’s method). This enrichment analysis provided an unbiased (data-driven) picture of which cell types might be linked to the GWAS phenotype. We used a window of 20 kb centered around each GWAS SNP to define GWAS positional candidates; this window prioritizes specificity (i.e. contains the most likely candidate genes) at the cost of sensitivity (many potential candidates will be missed). We then examined the literature to find other cell types frequently associated with the GWAS disease. Any cell types highlighted by either (1) the enrichment analysis or (2) the literature were considered as priority cell types. The majority of “priority” cell types for a particular GWAS were highlighted by both approaches.

In the second phase, we searched for genes located near GWAS SNPs that are associated with one of the priority cell types by CellMapper. For this phase, sensitivity was emphasized over specificity: we considered any genes in linkage disequilibrium with a GWAS SNP up to a maximum distance of 250 kb and selected all CellMap genes with an FDR ≤ 0.1.

#### Experimental validation of predicted GWAS candidate gene expression

Purified cell samples were isolated for qRT-PCR as follows: for murine immune cells, splenocytes were isolated from C57BL/6 wild-type mice. Cells were sorted by fluorescence-activated cell sorting (FACS) based on the following cell surface stains: B cells, CD3^−^ CD19^+^; NK cells, CD3^−^ CD19^−^ NK1.1^+^; dendritic cells, Lin^−^ (CD3, CD19) CD11b^+^ CD11c^+^ F4/80^−^; macrophage, Lin^−^ (CD3, CD19) CD11b^+^ F4/80^+^; neutrophils, CD11b^+^ Ly6G^+^; T cells, CD3^+^ CD19^−^. For other murine hematopoietic cells, 10–14-week old C57Bl/6 mouse bone marrow cells were isolated by crushing iliac crest bones, femurae, and tibiae in phosphate buffered saline (PBS) containing 5 % FCS and 2 mM EDTA. After red blood cell lysis, the remaining cells were stained with monoclonal antibodies and sorted by FACS as described in Pronk et al. [51]. For human immune cells, peripheral blood mononuclear cells were isolated from leukapheresis packs using a ficoll gradient. Cells were sorted by FACS based on the following cell surface stains: B cells, CD3^−^ CD19^+^; NK cells, CD3^−^ CD19^−^ CD56^+^; monocytes, Lin^−^ (CD3, CD19) CD14^+^; T cells, CD3^+^ CD19^−^. For solid tissue cells, HMEC-1 cells were obtained from Sean Colgan and grown in MCDB 131 (Gibco) supplemented with 10 % fetal bovine serum (Gibco), 10 mM L-glutamine (Gibco), 10 ng/mL mouse Epidermal Growth Factor (Peprotech), and 1 ug/mL Hydrocortisone (Sigma). Caco-2 BBe cells were obtained from Jerry Turner (University of Chicago) and grown in DMEM (Gibco) supplemented with 10 % fetal bovine serum. Two weeks before lysing cells for qRT-PCR, Caco-2 cells were plated on 0.4 um polycarbonate Transwell inserts (Corning) and grown with media changes three times per week. Primary epithelial organoids were generated from endoscopic biopsy samples of normal human duodenum and cultured according to Sato et al. [52].

For qRT-PCR, RNA was extracted from the purified cell populations using the RNeasy micro kit (Qiagen), then converted to first strand complementary DNA using Superscript III reverse transcriptase (Invitrogen). Quantitative PCR was performed on a BioRad C1000 Thermal Cycler with a CFX96 Real Time PCR Detection System using SYBR Green Master Mix (Invitrogen). Fold expression change was calculated using a variant of the ${2}^{-\varDelta \varDelta {C}_T}$ method for multiple reference genes [53]. We selected *OAZ1* and *SUMO2* as reference genes for mouse and *SUMO2* and *TBP* as reference genes for human. Calibrator samples were arbitrarily chosen as erythrocyte (Fig. 4b), organoid (Fig. 4d, left), and T cells (Fig. 4d, right). Primer sequences were designed using primer blast [54] and synthesized by Integrated DNA Technologies (Coralville, IA, USA).

To identify differential gene expression between cell types, we first tested which of three linear models best fit our data. The simplest model was that there is no difference in gene expression between cell types (the Null model). The next model was that there are gene expression differences between cell types, but not between negative control cell types (the Cell Class model). The final, and most complex, model was that there are gene expression differences between cell types regardless of class (the Independent Cell Type model). Negative control cell types were defined prior to analysis and were: GMP, Neu, M, cDC, B, T, and NK for *TRIM58*; T, NK, B, Mono, and HMEC-1 for *C1orf106*; and B, Mono, HMEC-1, Caco2, and Organoid for *KIF21B*. The simplest model was preferred unless a more complex model was a significantly better fit to the data (*p* ≤ 0.05, nested ANOVA F-test). The Cell Class model was the best fit for *TRIM58* and *C1orf106* and the Independent Cell Type model was the best fit for *KIF21B*. Once the model was chosen, we tested for differences between sample groups (either Independent Cell Types or Cell Classes) using Tukey’s honest significant difference test.

### Multiple-hypothesis testing

All *p* values were corrected for multiple hypothesis testing. FDR was used when our goal was to identify candidate cell type-enriched genes, as our conclusions would not change if a small subset of these predictions were false positives (Benjamini–Hochberg correction). Family-wise error rate *p* values were used when the results of a statistical test were interpreted directly and any false discoveries would alter the conclusions (Holm’s method).

## Additional files

Additional file 1: Description of CellMapper algorithm development and rationale for the SVD filter. (PDF 92 kb) Additional file 2:
*Schematic* of the CellMapper SVD filter and algorithm. CellMapper first performs an SVD of the microarray expression matrix to extract major components of variation (singular vectors). Then it re-weights the components of variation based on their estimated relevance to the query gene, with larger weights given to components that are tightly correlated with the query gene (e.g. “Component 3” is highly correlated with the query gene expression pattern and receives a large weight). Then the microarray data are reconstructed from the components using the estimated weights. The result of this SVD filter is to emphasize the components of variation that most distinguish the query gene and dampen components that are less relevant to the given query. After the SVD filtering process, genes are ranked based on the Pearson’s correlation of their transformed expression pattern to that of the query gene. (PDF 22 kb) Additional file 3: Algorithm development, part 1: Performance evaluation of five prospective algorithms using TiGER tissue genes as a gold standard [55], compared to the final algorithm *CellMapper*. Tukey boxplots show the change in area under the precision recall curve (AUPR) for each tissue, relative to the AUPR achieved by the best-performing prospective algorithm for that tissue. While all five prospective algorithms performed poorly relative to the others in several tissues, CellMapper achieved the highest AUPR in 25 out of 30 tissues and was always within 20 % AUPR of the best method. This analysis was for algorithm development (see Additional file 1): the prospective algorithms were not originally developed to identify cell type-enriched or tissue-enriched genes, but we tested them in this application because they have been effective using 1–2 query genes in other contexts, such as finding genes in co-regulated biological pathways (e.g. similar GO terms). *MEM* multi experiment matrix [44], *SPELL* Serial Patterns of Expression Levels Locator [45], *GR* Gene Recommender [56], *MI* mutual information. (PDF 11 kb) Additional file 4: Algorithm development, part 2: Parameter optimization for the CellMapper SVD filter, using test searches to find tissue-enriched genes as defined in the TiGER database [55]. **a** Evaluation of the free parameter, alpha. The SVD filter incorporates a free parameter, alpha, which allows the strength of the filter to be tuned, ranging in value from 1 (weak filter) to 0 (strong filter). Alpha values between 1 and 0.3 led to an increase in AUPR for 25 out of 30 tissues. An intermediate value of 0.5 was chosen for the final algorithm and this parameter was fixed prior to all analyses presented in the main text. **b** Evaluation of the query-driven weight term (QDW). The SVD filter also includes a term, abbreviated *QDW*, that decreases the weight of components in which the query genes are not well separated from the rest of the genome. The QDW term leads to an increase in performance beyond what is seen using the alpha scaling factor alone. ***, *p* < 10^−4^; Wilcoxon singed rank test. In both subfigures, AUPR was plotted relative to alpha = 1 and no query-driven weight term, which is approximately equivalent to Pearson’s correlation (it is equal to Pearson’s correlation with the low variance principle components filtered, see “Methods”). (PDF 20 kb) Additional file 5: Robustness of CellMapper to bias in dataset composition. Samples were drawn from the Lukk et al. [28] dataset in order to intentionally increase or decrease bias in sample composition and the effect on algorithm performance was quantified. **a** Sensitivity to adding redundant samples. CellMapper was applied, with and without the SVD filter, to search for tissue-specific genes using 500 randomly selected samples from the total microarray dataset, plus varying numbers of added “redundant samples.” For this analysis, “redundant samples” were selected from a subset of the data annotated as “blood,” “bone marrow,” and “mammary gland” because these three sample annotations are the most over-represented in the Lukk dataset, accounting for over half of all samples. While performance degraded when redundant samples were added without the SVD filter, CellMapper actually performed better and was able to benefit from the increase in sample size. **b** Sensitivity to removing relevant samples. Samples annotated as belonging to a specific tissue were removed from the Lukk dataset and CellMapper was applied to search this truncated dataset for genes expressed in the tissue with samples removed. This analysis was run separately for each of seven tissues (“bone,” “colon,” “kidney,” “liver,” “ovary,” “prostate,” and “skin”), and the mean change in AUPR across all tissues is reported. These tissues were analyzed because they represent an intermediate number of samples in the Lukk dataset (50–150 samples for each tissue or 1–3 % of the total). (PDF 19 kb) Additional file 6: Robustness of CellMapper to query gene choice; companion figure to Fig. 1b. To test the sensitivity of CellMapper to the choice of query gene, we repeated our analysis for kidney podocytes using ten distinct query genes (*MAFB* and the nine positive control genes in Additional file 13) and then assessed how well each analysis recovered an independent, experimentally-defined set of podocyte genes in mouse [23]. This plot shows the area under the precision-recall curve (AUPR) achieved when using each of the ten query choices. For comparison, we included a *dotted line* for the AUPR achieved by in silico nano-dissection when given all ten query genes at once (*light blue dotted line*; the area under the *light gray line* in Fig. 1b), or when given the original training set of 47 positive control and 97 negative control genes (*dark blue dotted line*; the area under the *dark gray line* in Fig. 1b). All ten single query gene searches for CellMapper resulted in a higher AUPR than in silico nano-dissection achieved when given all ten of these genes at once. *MAFB* was selected as the primary query gene for podocytes in this study (i.e. the *red line* in Fig. 1b) because it was used by the Genitourinary Developmental Molecular Anatomy Project [57] (GUDMAP) for all podocyte labeling and isolation in their large evaluation across kidney cell types. (PDF 20 kb) Additional file 7: CellMapper predictions for each of the 30 cell types. (XLSX 2677 kb) Additional file 8: Robustness of CellMapper to dataset size and composition. CellMapper was applied to predict genes expressed in each of the eight brain cell types, using random subsets of (*left*) the Allen Brain Atlas dataset or (*right*) an independent expression compendium of 1237 human brain samples drawn from the Lukk et al. [28] and Engreitz et al. [27] datasets (the “Affymetrix Brain Samples” compendium). The accuracy with which each search identified the experimentally-defined cell type genes in mice [3–6] was then quantified by the area under the precision-recall curve (AUPR). AUPR was calculated for 50 randomly sampled datasets of the indicated sample sizes and then mean AUPR was calculated. Results are reported as a *heatmap*, with all AUPRs scaled relative to the performance achieved when using the complete Allen Brain Atlas dataset for each cell type. The sensitivity to dataset abundance varied, with maximum AUPR being reached between 100 and 2000 microarray samples depending on the cell type. Overall, we conclude that a high quality, large, and uniformly collected dataset such as the Allen Brain Atlas is likely to allow for accurate predictions for a wider range of cell types. *Black squares* indicate that the AUPR was not significantly different from chance (Bonferroni corrected *p* value > 0.05; permutation test). (PDF 81 kb) Additional file 9: Brief overview of computational algorithms tested in Fig. 2. (PDF 100 kb) Additional file 10: Previous algorithms fail to identify established marker genes for four neural cell types; companion figure to Fig. 2b. This figure replicates Fig. 2a using in silico nano-dissection, DSA, and Pearson’s correlation—the best performing previous algorithms. *Dot charts* display the rank of classic cell-specific markers (positive controls) for the four neural cell types, as predicted by (**a**) in silico nano-dissection, (**b**) DSA, or (**c**) Pearson’s correlation. *Dots* are colored based on their known primary cell type of expression. *Dark gray shading* covers the area (rank list) required to identify all positive control genes for each cell type. Only CellMapper accurately identified classic marker genes for these cell types. (PDF 55 kb) Additional file 11: Performance evaluation of CellMapper and other computational methods to recover genes expressed in four major brain cell classes: neurons, astrocytes, oligodendrocytes, and microglia. Unlike the neural cell types examined in Fig. 2 and Additional file 16, these four cell classes are fairly common in the brain and have been successfully analyzed by previous computational algorithms. Each method was evaluated based on the recovery of an experimentally defined [6] set of cell type-enriched genes in mouse, as quantified by the area under the precision recall curve (AUPR). All methods show some resolution to resolve genes expressed in these cell types, but the best performance was consistently from CellMapper and in silico nano-dissection. (PDF 24 kb) Additional file 12: Table of query genes and expression datasets used for each cell type in this study. (PDF 97 kb) Additional file 13: Table of positive control cell type markers selected for Figs. 2 and 3. Five markers were chosen for each of the neuron subtypes and intestinal epithelial subtypes, four for NG2 Glia, and ten for every other cell type. Genes in bold were used as negative control markers for non-target cell types in Fig. 3. (PDF 152 kb) Additional file 14: Table of genes within GWAS loci predicted for expression in relevant cell types, focusing on loci associated with red blood cell phenotypes, platelet phenotypes, or inflammatory bowel disease (IBD). (XLSX 24 kb) Additional file 15: Human disease genes are enriched in the top CellMapper predictions; companion figure to Fig. 4. Enrichment of genes linked to human genetic disorders (OMIM) or human GWAS phenotypes (NHGRI) in the top CellMapper predictions. (**a**, **c**) Overall enrichment of human disease genes within the top CellMapper predictions across all 30 cell types, as a function of the gene rank cutoff. (**b**, **d**) Enrichment of genes linked to an individual OMIM disorder or GWAS phenotype within the top 200 genes predicted for a given cell type. All cell type-disease enrichments that reached statistical significance are shown. In panel (**d**), a more permissive FDR cutoff of 0.2 was selected to favor sensitivity in identifying potentially informative disease-cell type associations. Note that at this cutoff, one in five associations are expected to occur by chance and any conclusions should be interpreted appropriately. *Syn.* syndrome, *EKVP* erythrokeratodermia variabilis et progressiva, *ARVD* arrhythmogenic right ventricular dysplasia, *SED* spondyloepiphyseal dysplasia, *SCID* severe combined immunodeficiency, *FCAS* familial cold autoinflammatory syndrome, *CMT* Charcot-Marie-Tooth disease, *MADD* multiple Acyl-CoA dehydrogenase deficiency. (PDF 176 kb) Additional file 16: Robustness of CellMapper to query gene choice; companion figure to Fig. 2a. To test the sensitivity of CellMapper to the choice of query gene, we repeated our analysis for the three neuron subtypes and NG2 glia using each of the classic cell markers (positive controls) as query genes. *Dot charts* display the rank of the non-query gene classic markers within CellMapper’s predictions for each cell type. *Dots* are colored based on their known primary cell type of expression. *Dark gray shading* covers the area (rank list) required to identify all positive control genes for each cell type. Genes with promoters that have been used to drive cell-specific expression in mice (i.e. cell-specific reporter mouse strains available from cre.jax.org) are highlighted in *bold* with an asterisk under the “Query Gene” column. These genes have well-established expression patterns in the selected cell type and generally performed well as query genes. Many of the other classic cell markers have alternative sites of expression and were less effective as query genes. For instance, *SLC18A2* is expressed strongly in both serotonergic and noradrenergic neurons and returned markers expressed in both cell types. Factors to consider when choosing query genes for other cell types are described in the CellMapper R Package vignette (http://bioconductor.org/packages/CellMapper/). (PDF 69 kb)
