# Supplementary material for: CellMapper: rapid and accurate inference of gene expression in difficult-to-isolate cell types
Source: Genome Biol. 2016 Sep 29;17:201. doi: 10.1186/s13059-016-1062-5 (PMC5043525; doi:10.1186/s13059-016-1062-5)
Supplement: Additional file 1: — Description of CellMapper algorithm development and rationale for the SVD filter. (PDF 92 kb) [file 13059_2016_1062_MOESM1_ESM.pdf]

Additional file 1: Algorithm Development and Rationale for the Singular Value Decomposition (SVD) Filter

Most experimental techniques to isolate or otherwise manipulate specific cell types require only one or two marker genes. On the other hand, recent computational deconvolution algorithms with the best performance for rare and difficult-to-isolate cell types [1, 2] require very large training sets of positive and negative control genes to define any cell type. This limitation makes these algorithms difficult to apply, and prevents their application to cell types where only a few cell-specific marker genes are known. To develop a versatile and broadly applicable approach to find cell type-enriched genes, we felt it essential that the algorithm be accurate using only a small number of query genes (1-2 genes). This section describes the development and rationale for the CellMapper algorithm; a more thorough comparison between CellMapper and existing computational deconvolution algorithms can be found in the main text.

For all analyses presented in this section, we focus on tissue-specific gene expression (e.g. liver, intestine, heart) rather than cell type-specific expression. The reason for this choice is that there are large catalogs of tissue-specific genes to serve as a “gold standard” for performance evaluation [3]. This strategy also allowed us to perform algorithm development and optimization using an independent test case (tissue-specific expression), and fix all algorithm parameters before moving on to our primary interest (cell type-specific expression).

Initial Evaluation of Gene Co-Expression Algorithms

As a first attempt to establish a method that is accurate using only 1-2 marker genes, we tested several algorithms that were originally developed to find genes in co-regulated biological pathways (e.g. genes associated with the same GO terms) – GeneRecommender [4], MEM [5], and SPELL [6] – as well as mutual information. Each of these algorithms are compatible with small training sets (1-2

query genes) and have been demonstrated to identify genes in similar biological pathways; we hypothesized that one of these alternative algorithms might also be effective when applied to cell types.

Each prospective algorithm was tested against a gold standard of tissue-specific genes defined in the TiGER (Tissue-specific Gene Expression and Regulation) database [3], using a performance evaluation methodology similar to Hibbs et al. [6]. RefSeq IDs for all TiGER tissue genes were downloaded from the TiGER website [3], and mapped to Entrez IDs using biomaRt [7]. We performed individual query-driven searches using every possible combination of 2 from the top 20 genes classified as most tissue-enriched according to TiGER. For each algorithm, every search resulted in a list of all genes ranked from predicted most tissue-specific to least tissue-specific. We then calculated average gene rank across the lists generated by each query gene pair (excluding the query genes), producing a master list for each TiGER tissue and each algorithm, ordered from best average rank to worst. These lists were used to calculate precision (the number of TiGER genes identified at a given rank divided by the total number of genes identified at the same rank) and recall (the number of TiGER genes identified at a given rank divided by the total number of TiGER genes).

Unfortunately, none of the newer algorithms provided a consistent performance increase compared to even the simplest possible approach: Pearson's correlation. While each strongly outperformed correlation in some tissues, they all performed very poorly in many others (Additional file 3). Overall, the relative performance of the five algorithms was highly variable between tissues, with no single algorithm performing well across the board. This lead us to test alternative strategies to increase the sensitivity of Pearson's correlation, and we found success when filtering the data based on singular value decomposition (SVD), as described below.

Rationale for the SVD Filter

Singular value decomposition (SVD; also related to principal component analysis) of an expression matrix is the linear transformation of the original m genes by n arrays into an uncorrelated set of “eigengenes” and “eigenarrays” [8] given by:

$$X_{m\times n} = U_{m\times n}\Sigma_{n\times n}V_{n\times n}^{T}$$

where X is the expression matrix; U and V contain the eigenarrays (right-singular vectors) and corresponding eigengenes (left-singular vectors) of X, respectively; and Σ contains the singular values of X, or the relative importance (variance explained) of each eigenvector in the original expression matrix. SVD is widely used in genomics data analysis because the eigengenes and eigenarrays often have a biological interpretation. For instance, in the Lukk, et al. (2010) dataset used in this study, the first eigengene distinguishes hematopoietic from solid tissue samples [9], and the first eigenarray explains the corresponding genomic expression changes that accompany hematopoiesis.

While the top eigenvectors represent the strongest signals from the original expression matrix, they are not the most informative for every biological question. For instance, in an SVD analysis of yeast cell cycle microarrays, the first eigenvector explained over 90% of the gene expression data, yet the second and third eigenvectors contained most of the oscillating cell cycle gene expression signal [8]. The first eigenvectors can also relate to systematic technical noise such as lab effects [8, 10]. Finally, the strongest signals in a large meta-analysis of diverse samples will be dominated by the types of experiments performed most often in the literature; almost a third of the Lukk, et al. (2010) dataset contains microarrays from breast or breast cancer [9]. This sampling bias will disproportionately impact the first eigenvectors, while later eigenvectors may contain relevant information from biological conditions sampled less frequently. To increase the influence of potentially informative signatures from

the later eigenvectors, we filtered the data by adjusting the relative weight of each eigenvalue.

SVD Filter, Part 1: Flattening the Eigenvalues

One possibility would be to posit that each eigenvector has an equal chance of being informative, and weight all eigenvectors equally. The gene co-expression algorithm SPELL effectively takes this approach [6], by examining correlations between genes in eigenarray space. However, earlier eigenvectors contain a greater signal to noise ratio, and so weighting them equally with the lower (and noisier) eigenvectors may result in overemphasis of noise in the later eigenvectors. Therefore, we examined filters of the form:

$$\sigma_k' = \sigma_k^{\alpha}$$

which varies smoothly between no filter ($\alpha = 1$) to completely equalized eigenvalues ($\alpha = 0$; comparable to SPELL). Additional file 3a shows how AUPR varies as a function of α. The vast majority of tissues show an increase in AUPR for most values of α, and many demonstrate an increase in AUPR even as α approaches 0. We selected α to be 0.5 because this resulted in an improved AUPR for 25 out of 30 tissues ($p = 3.5 \times 10^{-7}$, Wilcoxon signed rank test), and never lead to a substantial decrease.

SVD Filter, Part 2: Filtering Eigenvectors that do not Differentiate the Query Genes

The above filter assumes that there is no way to identify which eigenvectors will best distinguish genes expressed in a given cell type. However, as we are defining cell type genes based on their similarity to

a set of query genes, we can expect that the most informative eigenvectors will be those where the query genes are well separated from the rest of the genome. Therefore, we apply a soft filter to the eigenvectors, multiplying each eigenvalue by a weight that increases as the query genes stand out from other genes:

$$w_k = \sum_{g \in (query\ genes)} \tanh\left(u_k^g\right)$$

where u_k^g is the loading of gene g in singular vector k, normalized so that u_k has a mean of 0 across all genes with a standard deviation of 1. This weight plateaus when the query genes are at least a standard deviation away from the mean value for an eigenvector, but approaches 0 as the query genes tend towards the mean. Additional file 3b shows that this query-driven weighting produces an increase in AUPR for almost all tissues regardless of the value of α ($p = 4.4 \times 10^{-4}$ for α = 1, $p = 9.3 \times 10^{-4}$ for α = 0.5; Wilcoxon signed rank test). After establishing these two suitable filters for ranking of tissue-specific genes, the same filters were applied to the identification of cell-type specific genes.

1. Ju W, Greene CS, Eichinger F, Nair V, Hodgin JB, Bitzer M, Lee Y-S, Zhu Q, Kehata M, Li M, Jiang S, Rastaldi MP, Cohen CD, Troyanskaya OG, Kretzler M: **Defining cell-type specificity at the transcriptional level in human disease.** *Genome Res* 2013, **23**:1862–73.

2. Chikina MD, Huttenhower C, Murphy CT, Troyanskaya OG: **Global prediction of tissue-specific gene expression and context-dependent gene networks in Caenorhabditis elegans.** *PLoS Comput Biol* 2009, **5**:e1000417.

3. Liu X, Yu X, Zack DJ, Zhu H, Qian J: **TiGER: a database for tissue-specific gene expression and regulation.** *BMC Bioinformatics* 2008, **9**:271.

4. Owen AB, Stuart J, Mach K, Villeneuve AM, Kim S: **A gene recommender algorithm to identify coexpressed genes in C. elegans.** *Genome Res* 2003, **13**:1828–37.

5. Adler P, Kolde R, Kull M, Tkachenko A, Peterson H, Reimand J, Vilo J: **Mining for coexpression across hundreds of datasets using novel rank aggregation and visualization methods.** *Genome Biol* 2009, **10**:R139.

6. Hibbs M a, Hess DC, Myers CL, Huttenhower C, Li K, Troyanskaya OG: **Exploring the functional**

landscape of gene expression: directed search of large microarray compendia. *Bioinformatics* 2007, **23**:2692–9.

7. Kasprzyk A: **BioMart: driving a paradigm change in biological data management.** *Database (Oxford)* 2011, **2011**:bar049.

8. Alter O, Brown PO, Botstein D: **Singular value decomposition for genome-wide expression data processing and modeling.** *Proc Natl Acad Sci U S A* 2000, **97**:10101–6.

9. Lukk M, Kapushesky M, Nikkilä J, Parkinson H, Goncalves A, Huber W, Ukkonen E, Brazma A: **A global map of human gene expression**. *Nat Biotechnol* 2010, **28**:322–4.

10. Nielsen TO, West RB, Linn SC, Alter O, Knowling MA, Connell JXO, Zhu S, Fero M, Sherlock G, Pollack JR, Brown PO, Botstein D, Rijn M Van De: **Mechanisms of disease Molecular characterisation of soft tissue tumours : a gene expression study**. 2002, **359**.
