# Supplementary figures and images for: CellMapper: rapid and accurate inference of gene expression in difficult-to-isolate cell types
Source: Genome Biol. 2016 Sep 29;17:201. doi: 10.1186/s13059-016-1062-5 (PMC5043525; doi:10.1186/s13059-016-1062-5)

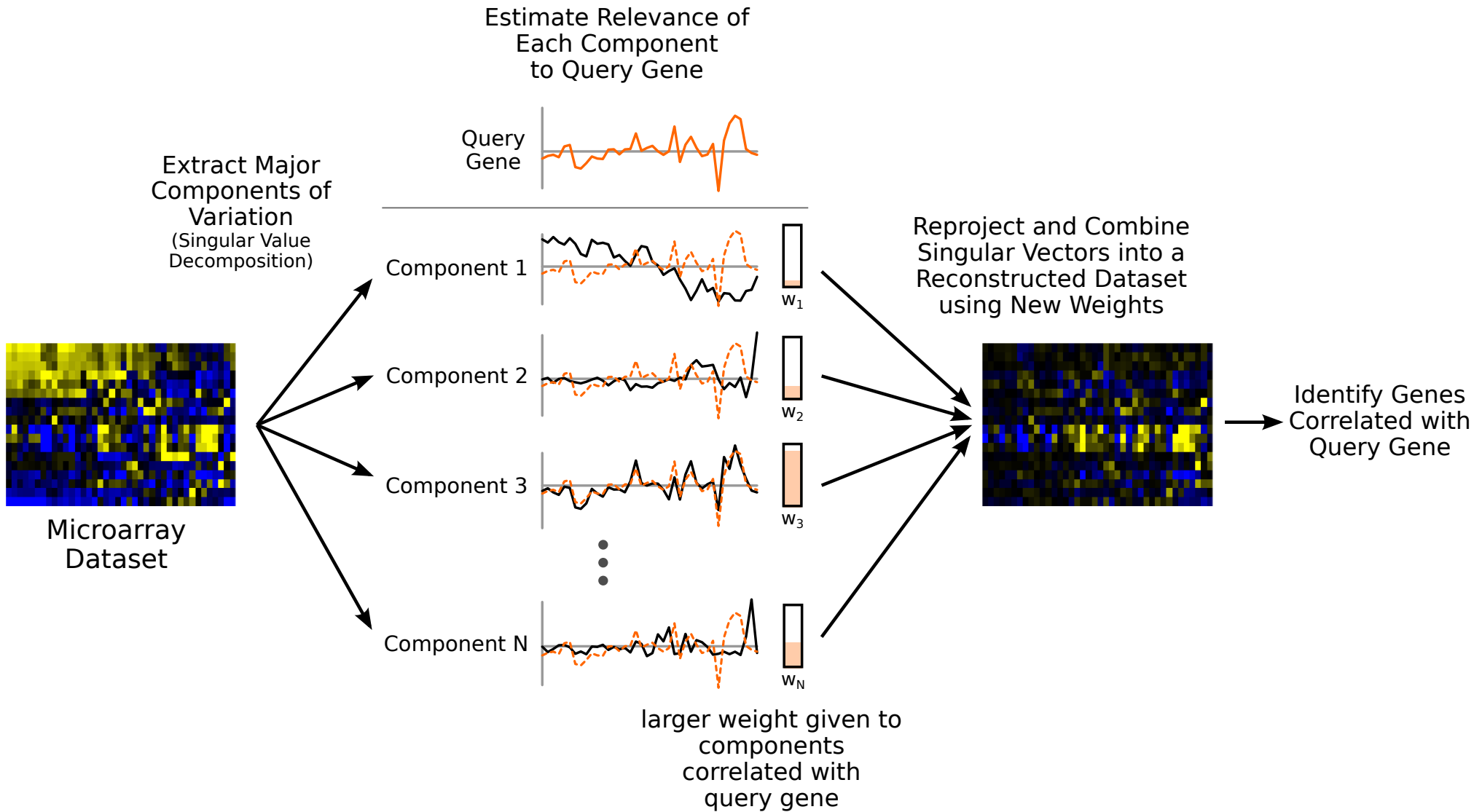

Supplement: Additional file 2: — Schematic of the CellMapper SVD filter and algorithm. CellMapper first performs an SVD of the microarray expression matrix to extract major components of variation (singular vectors). Then it re-weights the components of variation based on their estimated relevance to the query gene, with larger weights given to components that are tightly correlated with the query gene (e.g. “Component 3” is highly correlated with the query gene expression pattern and receives a large weight). Then the microarray data are reconstructed from the components using the estimated weights. The result of this SVD filter is to emphasize the components of variation that most distinguish the query gene and dampen components that are less relevant to the given query. After the SVD filtering process, genes are ranked based on the Pearson’s correlation of their transformed expression pattern to that of the query gene. (PDF 22 kb) [file 13059_2016_1062_MOESM2_ESM.pdf]

Fraction AUPR  
(relative to best performing  
prospective algorithm)

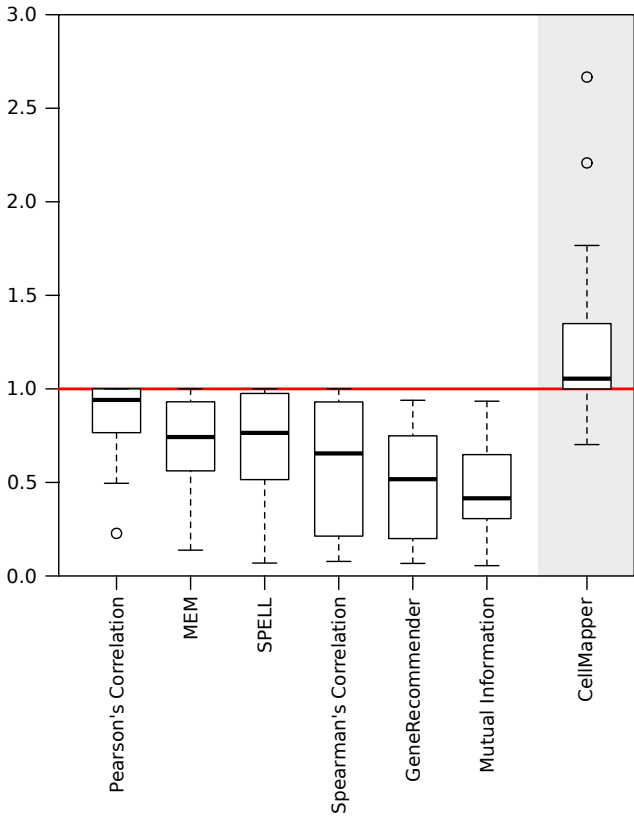

Supplement: Additional file 3: — Algorithm development, part 1: Performance evaluation of five prospective algorithms using TiGER tissue genes as a gold standard [55], compared to the final algorithm CellMapper. Tukey boxplots show the change in area under the precision recall curve (AUPR) for each tissue, relative to the AUPR achieved by the best-performing prospective algorithm for that tissue. While all five prospective algorithms performed poorly relative to the others in several tissues, CellMapper achieved the highest AUPR in 25 out of 30 tissues and was always within 20 % AUPR of the best method. This analysis was for algorithm development (see Additional file 1): the prospective algorithms were not originally developed to identify cell type-enriched or tissue-enriched genes, but we tested them in this application because they have been effective using 1–2 query genes in other contexts, such as finding genes in co-regulated biological pathways (e.g. similar GO terms). MEM multi experiment matrix [44], SPELL Serial Patterns of Expression Levels Locator [45], GR Gene Recommender [56], MI mutual information. (PDF 11 kb) [file 13059_2016_1062_MOESM3_ESM.pdf]

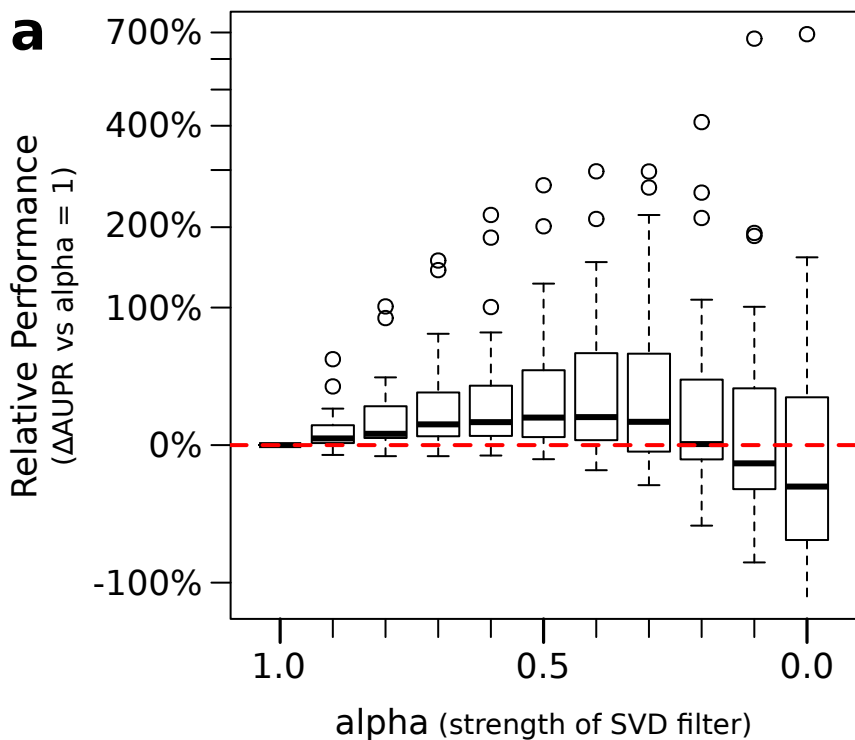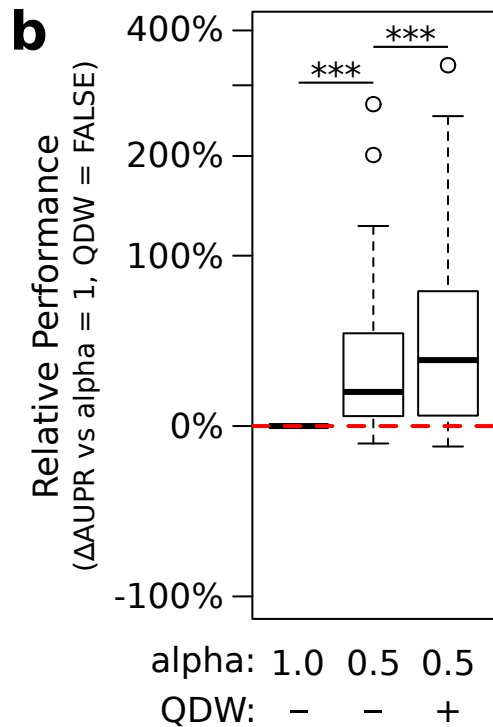

Supplement: Additional file 4: — Algorithm development, part 2: Parameter optimization for the CellMapper SVD filter, using test searches to find tissue-enriched genes as defined in the TiGER database [55]. a Evaluation of the free parameter, alpha. The SVD filter incorporates a free parameter, alpha, which allows the strength of the filter to be tuned, ranging in value from 1 (weak filter) to 0 (strong filter). Alpha values between 1 and 0.3 led to an increase in AUPR for 25 out of 30 tissues. An intermediate value of 0.5 was chosen for the final algorithm and this parameter was fixed prior to all analyses presented in the main text. b Evaluation of the query-driven weight term (QDW). The SVD filter also includes a term, abbreviated QDW, that decreases the weight of components in which the query genes are not well separated from the rest of the genome. The QDW term leads to an increase in performance beyond what is seen using the alpha scaling factor alone. ***, p < 10−4; Wilcoxon singed rank test. In both subfigures, AUPR was plotted relative to alpha = 1 and no query-driven weight term, which is approximately equivalent to Pearson’s correlation (it is equal to Pearson’s correlation with the low variance principle components filtered, see “Methods”). (PDF 20 kb) [file 13059_2016_1062_MOESM4_ESM.pdf]

**a**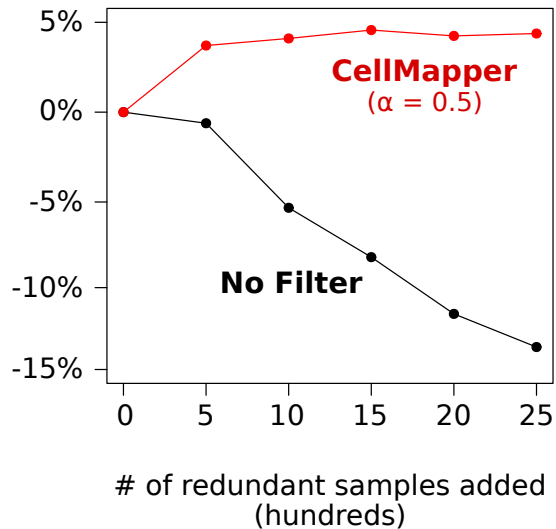**b**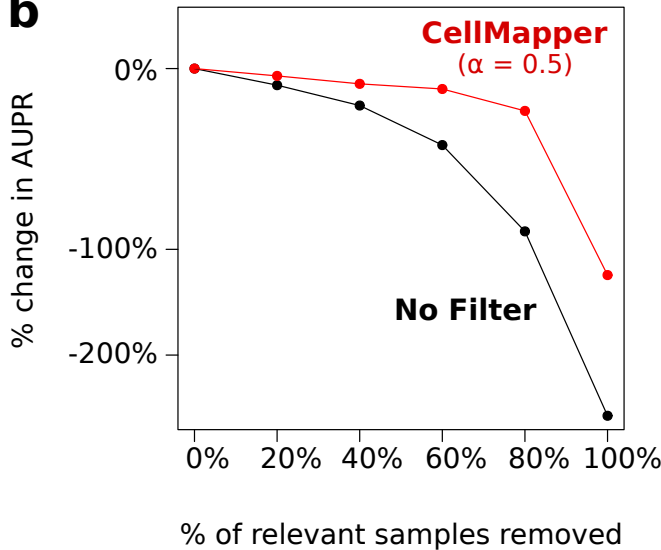

Supplement: Additional file 5: — Robustness of CellMapper to bias in dataset composition. Samples were drawn from the Lukk et al. [28] dataset in order to intentionally increase or decrease bias in sample composition and the effect on algorithm performance was quantified. a Sensitivity to adding redundant samples. CellMapper was applied, with and without the SVD filter, to search for tissue-specific genes using 500 randomly selected samples from the total microarray dataset, plus varying numbers of added “redundant samples.” For this analysis, “redundant samples” were selected from a subset of the data annotated as “blood,” “bone marrow,” and “mammary gland” because these three sample annotations are the most over-represented in the Lukk dataset, accounting for over half of all samples. While performance degraded when redundant samples were added without the SVD filter, CellMapper actually performed better and was able to benefit from the increase in sample size. b Sensitivity to removing relevant samples. Samples annotated as belonging to a specific tissue were removed from the Lukk dataset and CellMapper was applied to search this truncated dataset for genes expressed in the tissue with samples removed. This analysis was run separately for each of seven tissues (“bone,” “colon,” “kidney,” “liver,” “ovary,” “prostate,” and “skin”), and the mean change in AUPR across all tissues is reported. These tissues were analyzed because they represent an intermediate number of samples in the Lukk dataset (50–150 samples for each tissue or 1–3 % of the total). (PDF 19 kb) [file 13059_2016_1062_MOESM5_ESM.pdf]

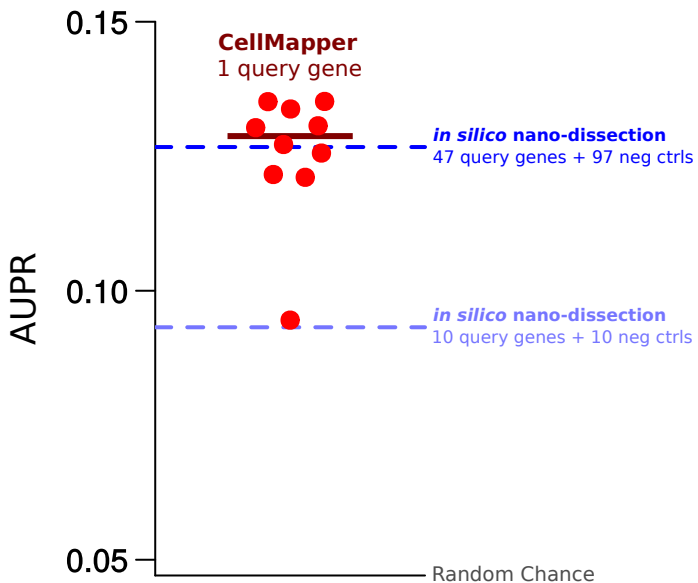

Supplement: Additional file 6: — Robustness of CellMapper to query gene choice; companion figure to Fig. 1b. To test the sensitivity of CellMapper to the choice of query gene, we repeated our analysis for kidney podocytes using ten distinct query genes (MAFB and the nine positive control genes in Additional file 13) and then assessed how well each analysis recovered an independent, experimentally-defined set of podocyte genes in mouse [23]. This plot shows the area under the precision-recall curve (AUPR) achieved when using each of the ten query choices. For comparison, we included a dotted line for the AUPR achieved by in silico nano-dissection when given all ten query genes at once (light blue dotted line; the area under the light gray line in Fig. 1b), or when given the original training set of 47 positive control and 97 negative control genes (dark blue dotted line; the area under the dark gray line in Fig. 1b). All ten single query gene searches for CellMapper resulted in a higher AUPR than in silico nano-dissection achieved when given all ten of these genes at once. MAFB was selected as the primary query gene for podocytes in this study (i.e. the red line in Fig. 1b) because it was used by the Genitourinary Developmental Molecular Anatomy Project [57] (GUDMAP) for all podocyte labeling and isolation in their large evaluation across kidney cell types. (PDF 20 kb) [file 13059_2016_1062_MOESM6_ESM.pdf]

# Allen Brain Atlas

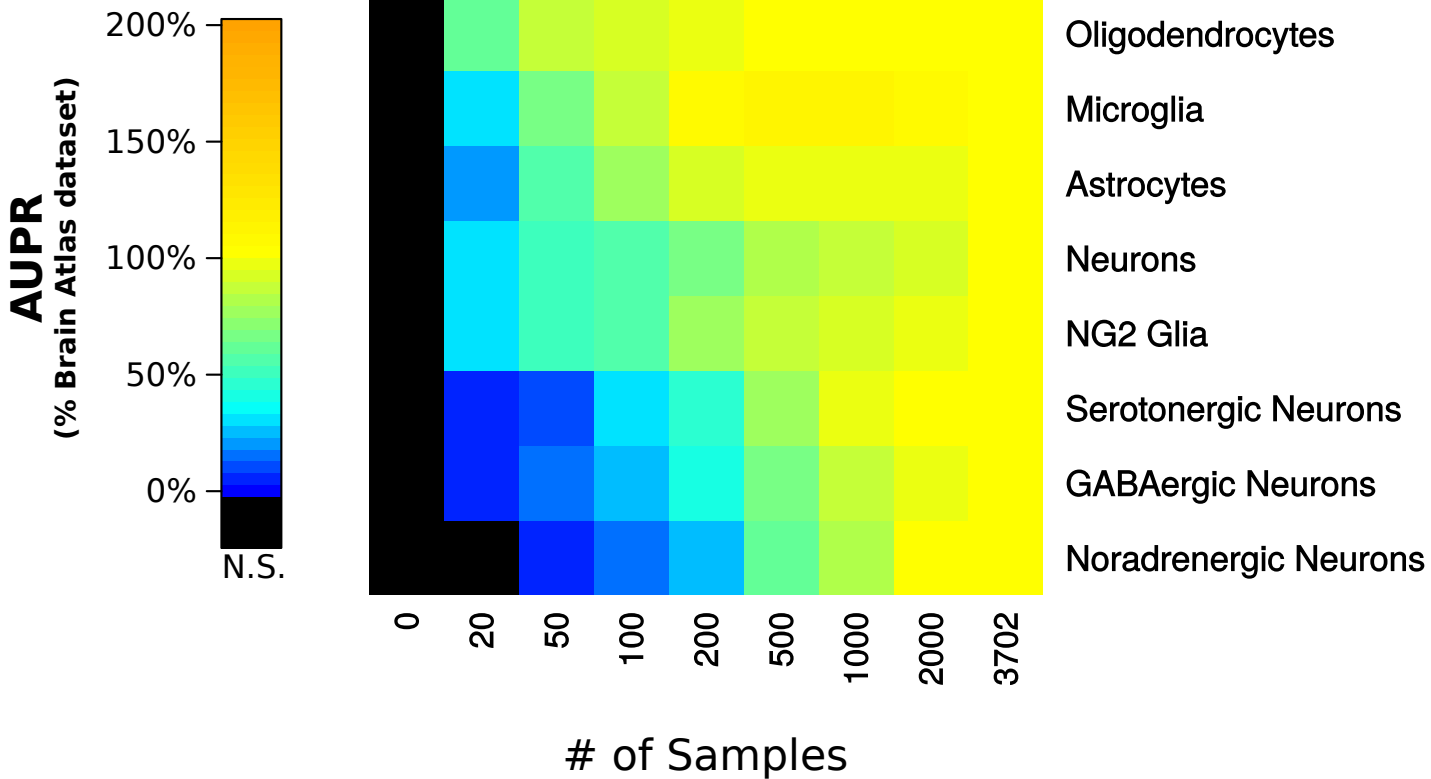

## Affymetrix Brain Samples

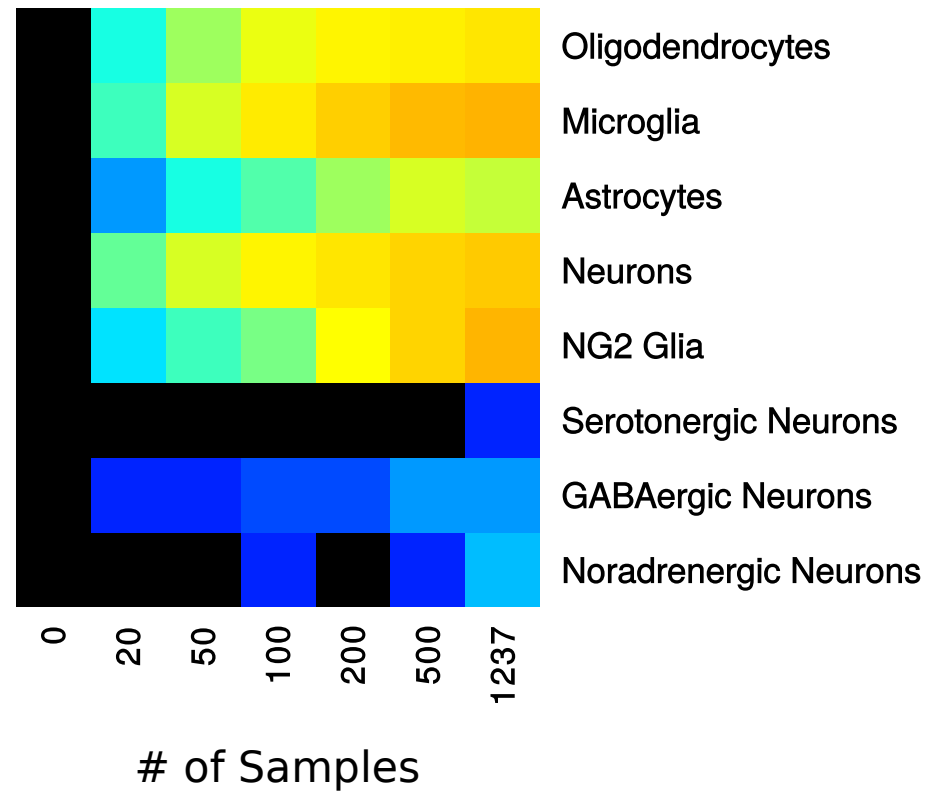

Supplement: Additional file 8: — Robustness of CellMapper to dataset size and composition. CellMapper was applied to predict genes expressed in each of the eight brain cell types, using random subsets of (left) the Allen Brain Atlas dataset or (right) an independent expression compendium of 1237 human brain samples drawn from the Lukk et al. [28] and Engreitz et al. [27] datasets (the “Affymetrix Brain Samples” compendium). The accuracy with which each search identified the experimentally-defined cell type genes in mice [3–6] was then quantified by the area under the precision-recall curve (AUPR). AUPR was calculated for 50 randomly sampled datasets of the indicated sample sizes and then mean AUPR was calculated. Results are reported as a heatmap, with all AUPRs scaled relative to the performance achieved when using the complete Allen Brain Atlas dataset for each cell type. The sensitivity to dataset abundance varied, with maximum AUPR being reached between 100 and 2000 microarray samples depending on the cell type. Overall, we conclude that a high quality, large, and uniformly collected dataset such as the Allen Brain Atlas is likely to allow for accurate predictions for a wider range of cell types. Black squares indicate that the AUPR was not significantly different from chance (Bonferroni corrected p value > 0.05; permutation test). (PDF 81 kb) [file 13059_2016_1062_MOESM8_ESM.pdf]

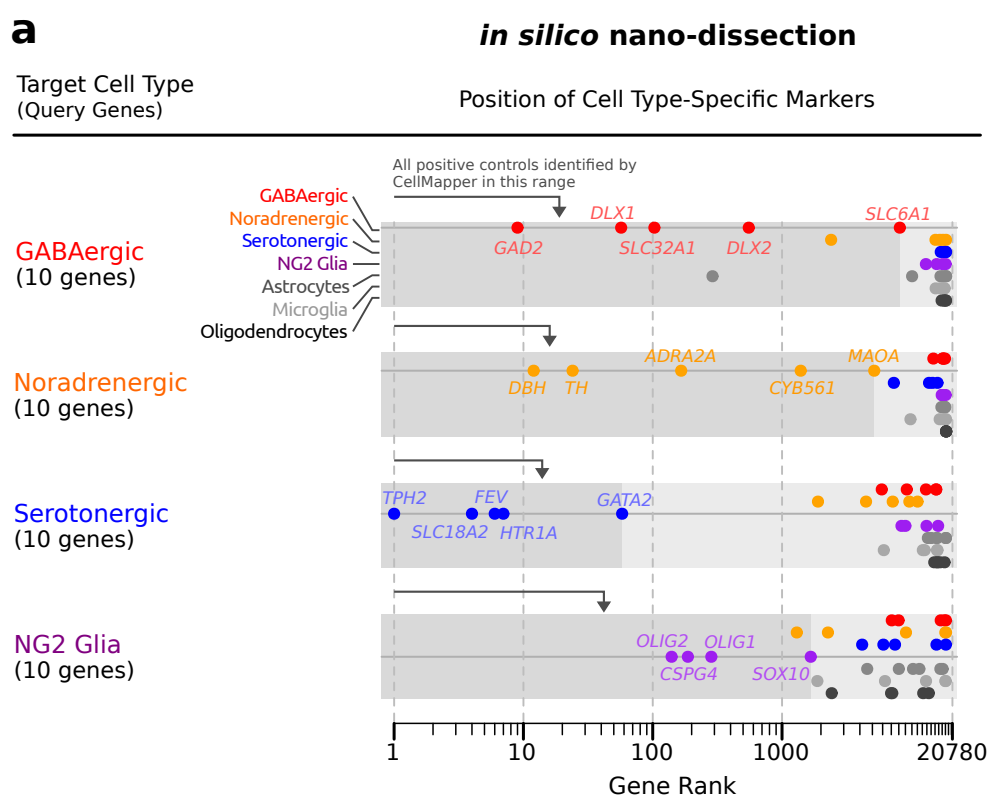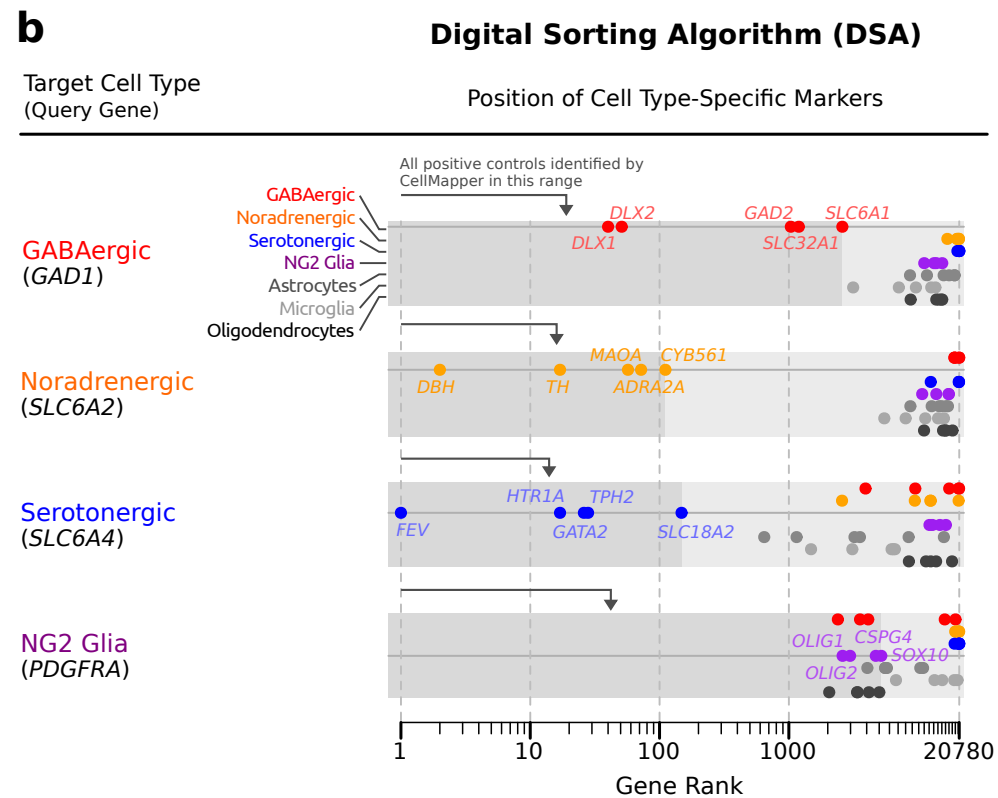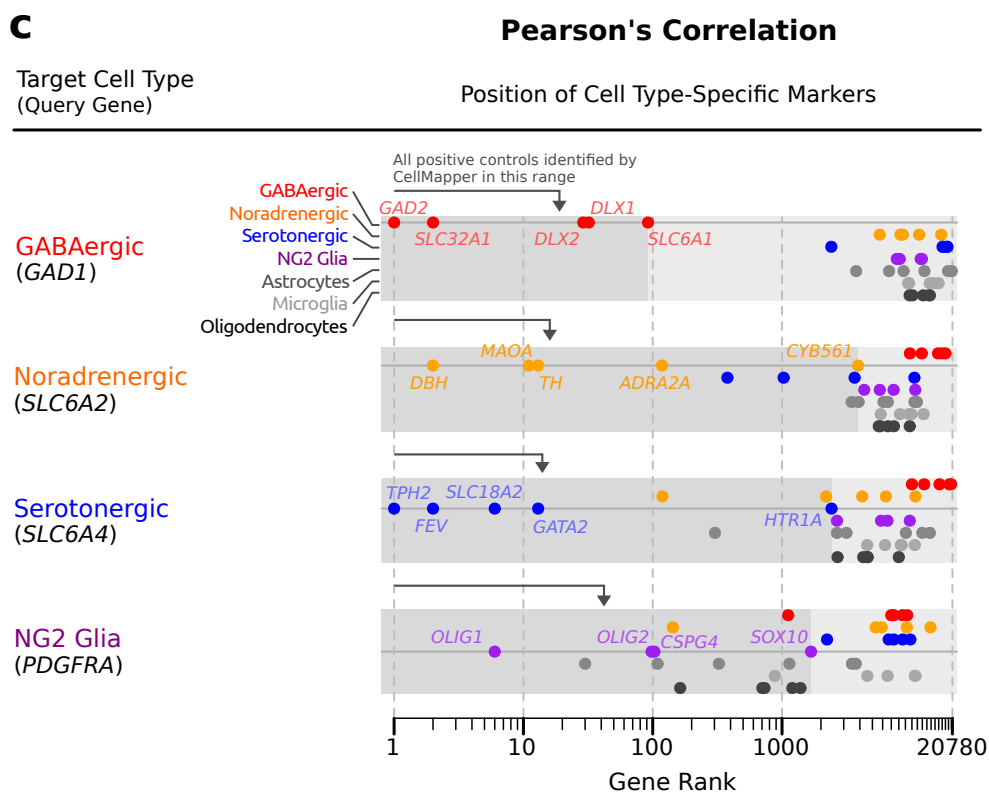

Supplement: Additional file 10: — Previous algorithms fail to identify established marker genes for four neural cell types; companion figure to Fig. 2b. This figure replicates Fig. 2a using in silico nano-dissection, DSA, and Pearson’s correlation—the best performing previous algorithms. Dot charts display the rank of classic cell-specific markers (positive controls) for the four neural cell types, as predicted by (a) in silico nano-dissection, (b) DSA, or (c) Pearson’s correlation. Dots are colored based on their known primary cell type of expression. Dark gray shading covers the area (rank list) required to identify all positive control genes for each cell type. Only CellMapper accurately identified classic marker genes for these cell types. (PDF 55 kb) [file 13059_2016_1062_MOESM10_ESM.pdf]

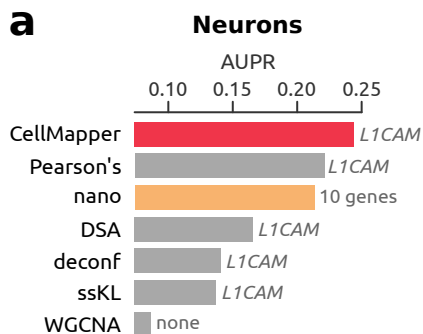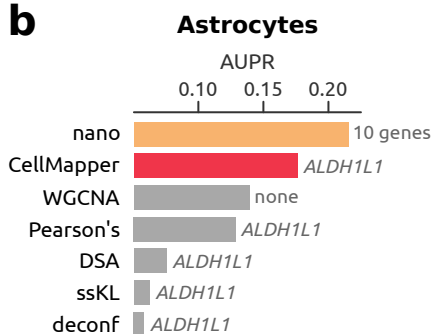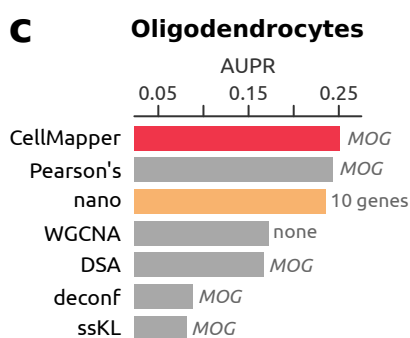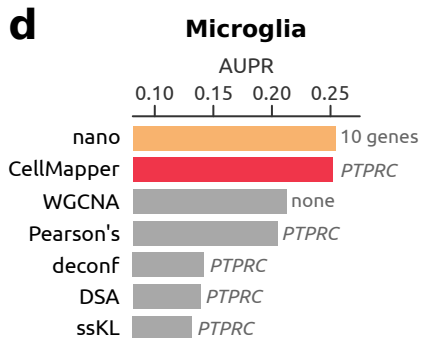

Supplement: Additional file 11: — Performance evaluation of CellMapper and other computational methods to recover genes expressed in four major brain cell classes: neurons, astrocytes, oligodendrocytes, and microglia. Unlike the neural cell types examined in Fig. 2 and Additional file 16, these four cell classes are fairly common in the brain and have been successfully analyzed by previous computational algorithms. Each method was evaluated based on the recovery of an experimentally defined [6] set of cell type-enriched genes in mouse, as quantified by the area under the precision recall curve (AUPR). All methods show some resolution to resolve genes expressed in these cell types, but the best performance was consistently from CellMapper and in silico nano-dissection. (PDF 24 kb) [file 13059_2016_1062_MOESM11_ESM.pdf]

**a** Human Genetic Disorders (OMIM)

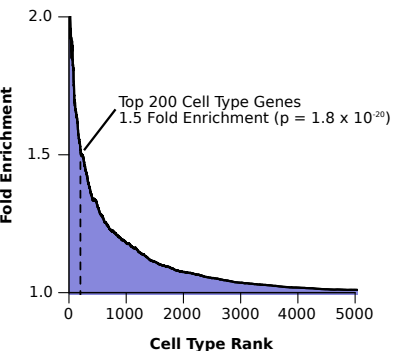

**b**

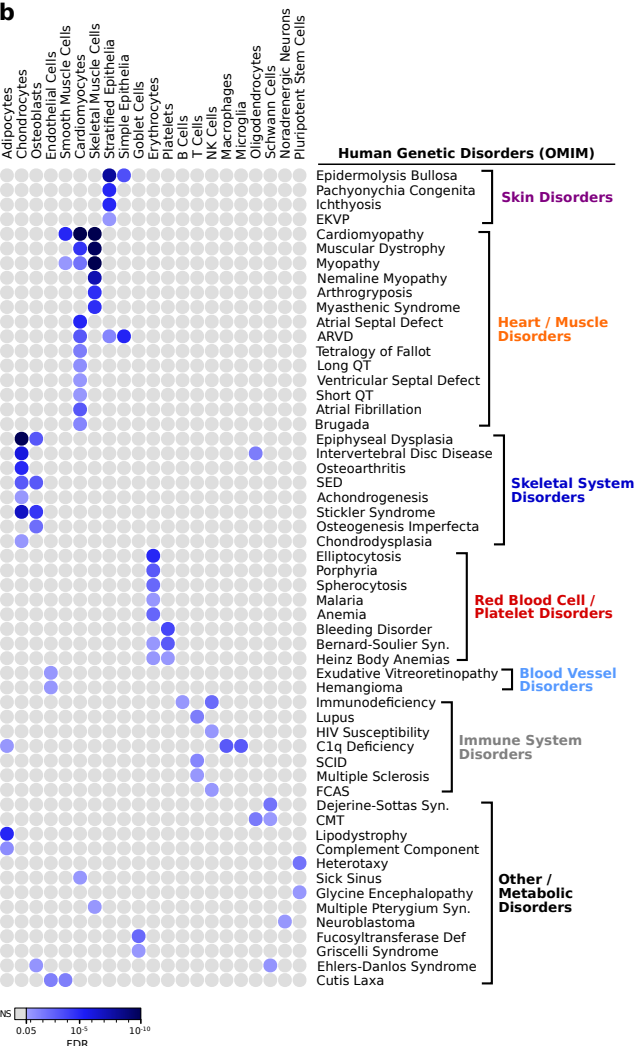

**c** GWAS Phenotypes (NHGRI)

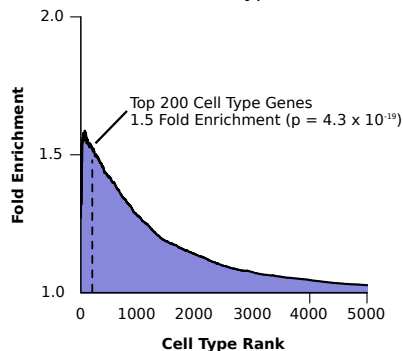

**d**

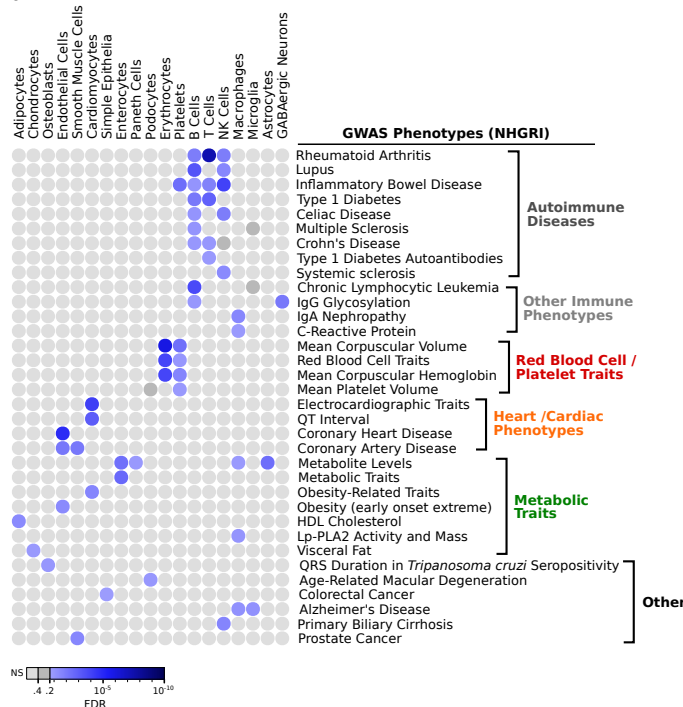

Supplement: Additional file 15: — Human disease genes are enriched in the top CellMapper predictions; companion figure to Fig. 4. Enrichment of genes linked to human genetic disorders (OMIM) or human GWAS phenotypes (NHGRI) in the top CellMapper predictions. (a, c) Overall enrichment of human disease genes within the top CellMapper predictions across all 30 cell types, as a function of the gene rank cutoff. (b, d) Enrichment of genes linked to an individual OMIM disorder or GWAS phenotype within the top 200 genes predicted for a given cell type. All cell type-disease enrichments that reached statistical significance are shown. In panel (d), a more permissive FDR cutoff of 0.2 was selected to favor sensitivity in identifying potentially informative disease-cell type associations. Note that at this cutoff, one in five associations are expected to occur by chance and any conclusions should be interpreted appropriately. Syn. syndrome, EKVP erythrokeratodermia variabilis et progressiva, ARVD arrhythmogenic right ventricular dysplasia, SED spondyloepiphyseal dysplasia, SCID severe combined immunodeficiency, FCAS familial cold autoinflammatory syndrome, CMT Charcot-Marie-Tooth disease, MADD multiple Acyl-CoA dehydrogenase deficiency. (PDF 176 kb) [file 13059_2016_1062_MOESM15_ESM.pdf]

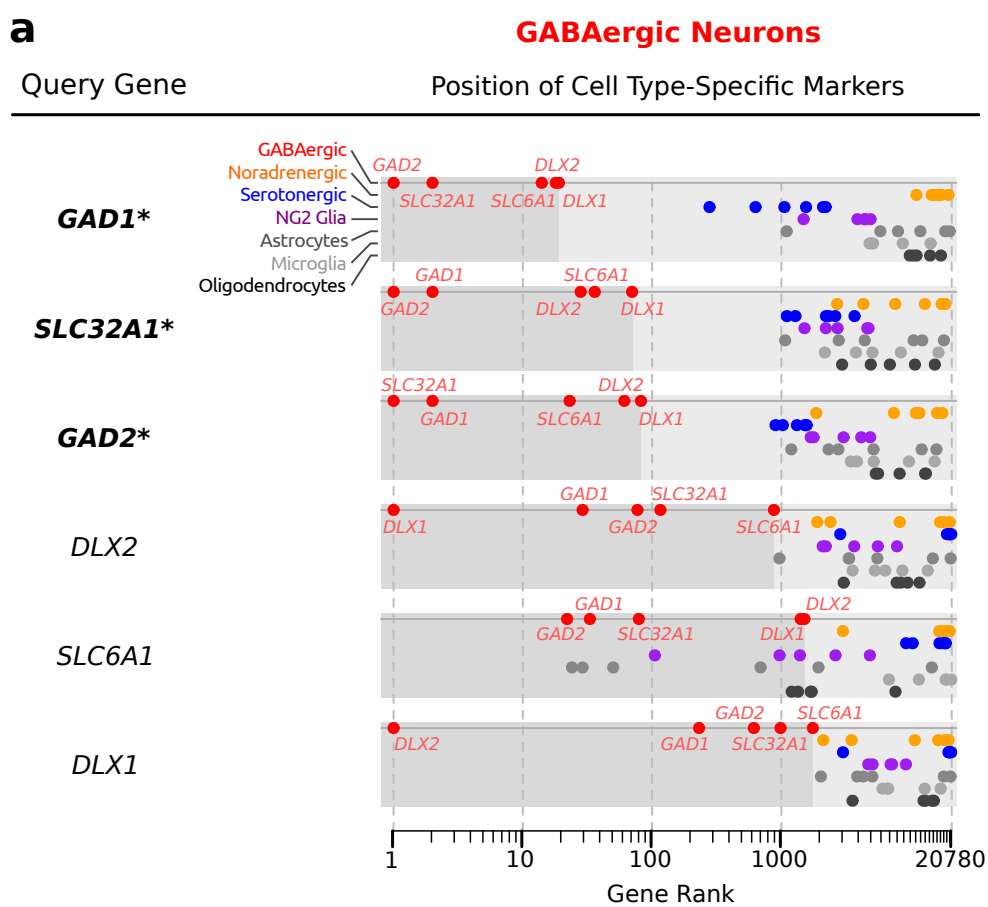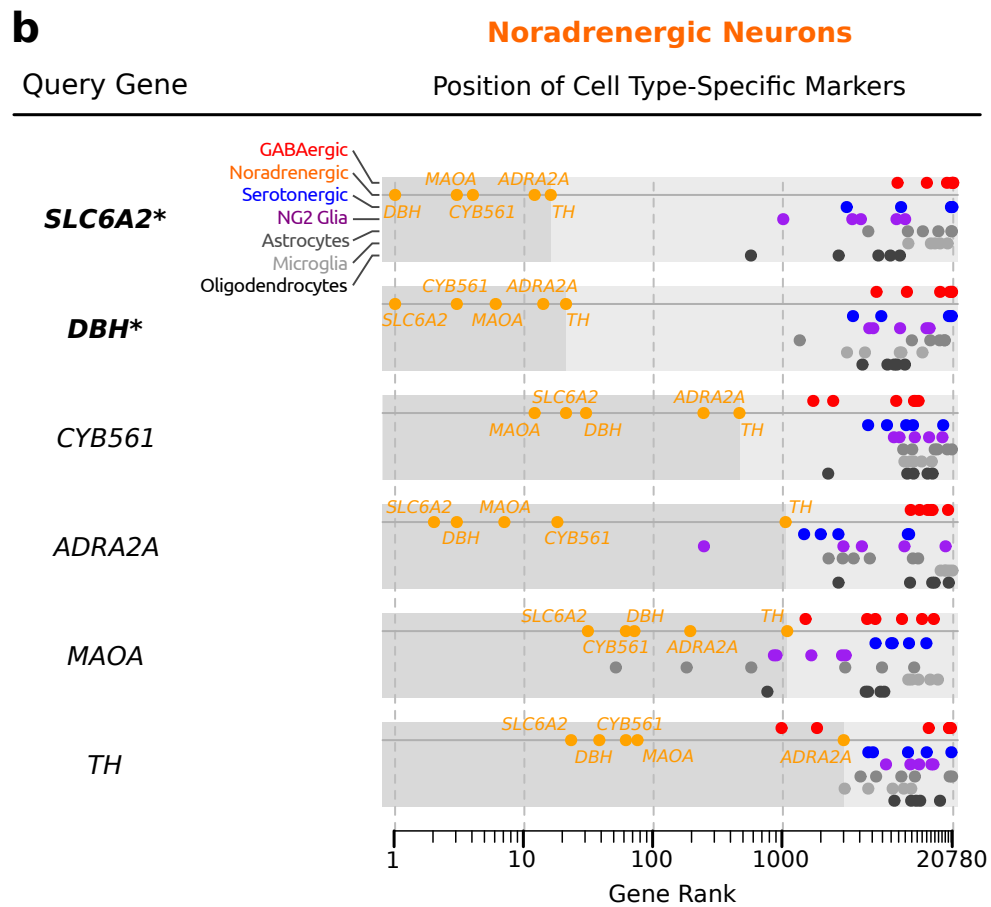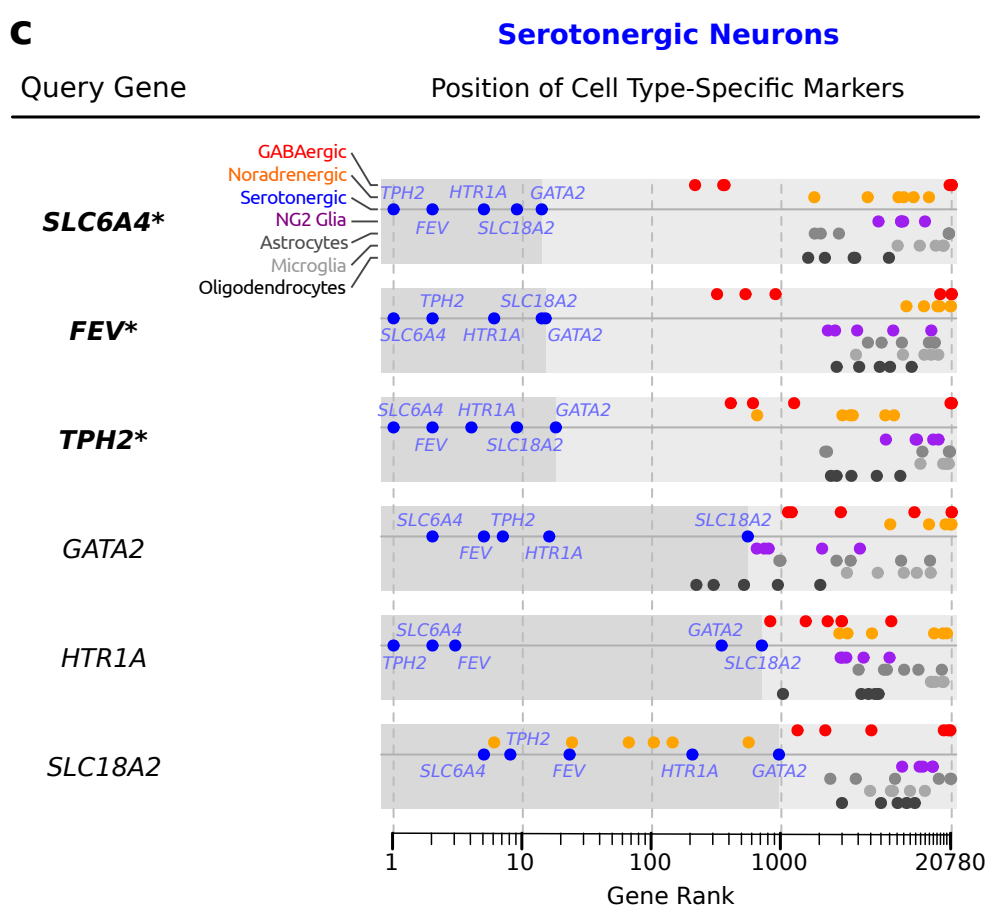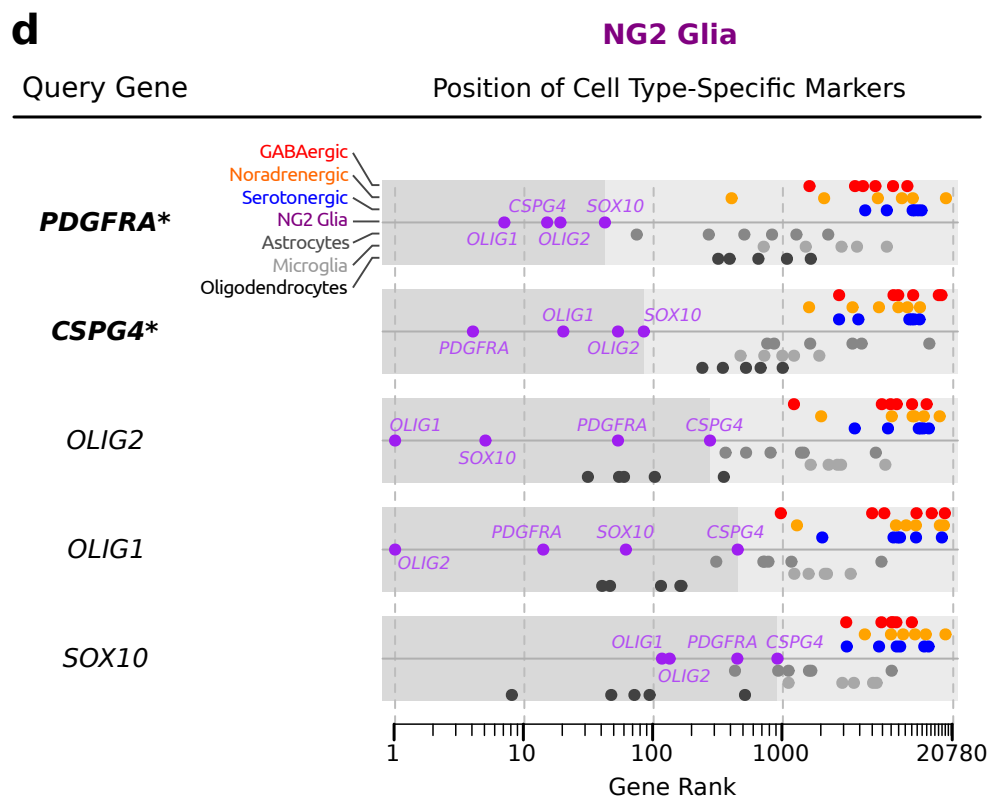

Supplement: Additional file 16: — Robustness of CellMapper to query gene choice; companion figure to Fig. 2a. To test the sensitivity of CellMapper to the choice of query gene, we repeated our analysis for the three neuron subtypes and NG2 glia using each of the classic cell markers (positive controls) as query genes. Dot charts display the rank of the non-query gene classic markers within CellMapper’s predictions for each cell type. Dots are colored based on their known primary cell type of expression. Dark gray shading covers the area (rank list) required to identify all positive control genes for each cell type. Genes with promoters that have been used to drive cell-specific expression in mice (i.e. cell-specific reporter mouse strains available from cre.jax.org) are highlighted in bold with an asterisk under the “Query Gene” column. These genes have well-established expression patterns in the selected cell type and generally performed well as query genes. Many of the other classic cell markers have alternative sites of expression and were less effective as query genes. For instance, SLC18A2 is expressed strongly in both serotonergic and noradrenergic neurons and returned markers expressed in both cell types. Factors to consider when choosing query genes for other cell types are described in the CellMapper R Package vignette (http://bioconductor.org/packages/CellMapper/). (PDF 69 kb) [file 13059_2016_1062_MOESM16_ESM.pdf]
