# Supplementary material for: CellMapper: rapid and accurate inference of gene expression in difficult-to-isolate cell types
Source: Genome Biol. 2016 Sep 29;17:201. doi: 10.1186/s13059-016-1062-5 (PMC5043525; doi:10.1186/s13059-016-1062-5)
Supplement: Additional file 9: — Brief overview of computational algorithms tested in Fig. 2. (PDF 100 kb) [file 13059_2016_1062_MOESM9_ESM.pdf]

Additional file 10: Brief Overview of Computational Deconvolution Algorithms

In Figure 2b-e, Additional file 11, and Additional file 12, we compared CellMapper to several existing computational algorithms. These approaches are each in some ways distinct from CellMapper:

- *In silico* nano-dissection [1] is a method aimed at identifying cell type-enriched genes similar to CellMapper, but it uses a machine learning algorithm that requires a large training set of positive and negative control genes for each cell type. It is difficult to curate a large training set of query genes for the four brain cell types we investigated, but we tested the method using the most careful list of query genes we could identify (see Methods).

- Weighted gene co-expression network analysis (WGCNA) is an unsupervised clustering method that has been shown to uncover gene clusters related to cell type-specific expression in the Allen Brain Atlas dataset [2] and other microarray datasets [3, 4] from brain. This algorithm does not require (or accept) a training set, and as a result cannot be targeted at particular cell types and may not be suitable for rare cell types that do not provide a dominant expression signal in the data.

- All marker-directed computational deconvolution algorithms in the *CellMix* [5] R package: deconf [6], the digital sorting algorithm (DSA) [7], and semi-supervised nonnegative matrix factorization (ssNMF) [8]. These algorithms were each motivated by a distinct biological problem from CellMapper: in diseases where the proportion of different cell types varies according to disease state (e.g. cancer, Huntington's disease), these methods can distinguish between changes in gene expression caused by changes in cell type frequency from those caused by altered gene expression within the individual cell types. To address this problem, these algorithms must estimate the *total* (rather than *relative*) expression level of every gene in each cell type, and estimate this separately for case and control groups. CellMapper does not

attempt to extract so much information from a dataset, but rather is focused on identifying which genes are consistently enriched in one cell type relative to others. We show that our focused strategy is much more sensitive for this application, especially for rare and difficult-to-isolate cell type.

1. Ju W, Greene CS, Eichinger F, Nair V, Hodgin JB, Bitzer M, Lee Y-S, Zhu Q, Kehata M, Li M, Jiang S, Rastaldi MP, Cohen CD, Troyanskaya OG, Kretzler M: **Defining cell-type specificity at the transcriptional level in human disease.** *Genome Res* 2013, **23**:1862–73.

2. Hawrylycz MJ, Lein ES, Guillozet-Bongaarts AL, Shen EH, Ng L, Miller J a, van de Lagemaat LN, Smith K a, Ebbert A, Riley ZL, Abajian C, Beckmann CF, Bernard A, Bertagnolli D, Boe AF, Cartagena PM, Chakravarty MM, Chapin M, Chong J, Dalley R a, Daly BD, Dang C, Datta S, Dee N, Dolbeare T a, Faber V, Feng D, Fowler DR, Goldy J, Gregor BW, et al.: **An anatomically comprehensive atlas of the adult human brain transcriptome.** *Nature* 2012, **489**:391–9.

3. Oldham MC, Konopka G, Iwamoto K, Langfelder P, Kato T, Horvath S, Geschwind DH: **Functional organization of the transcriptome in human brain.** *Nat Neurosci* 2008, **11**:1271–82.

4. Lui JH, Nowakowski TJ, Pollen AA, Javaherian A, Kriegstein AR, Oldham MC: **Radial glia require PDGFD-PDGFRb signalling in human but not mouse neocortex**. *Nature* 2014.

5. Gaujoux R, Seoighe C: **CellMix: A comprehensive toolbox for gene expression deconvolution**. *Bioinformatics* 2013, **29**:2211–2212.

6. Repsilber D, Kern S, Telaar A, Walzl G, Black GF, Selbig J, Parida SK, Kaufmann SHE, Jacobsen M: **Biomarker discovery in heterogeneous tissue samples -taking the in-silico deconfounding approach.** *BMC Bioinformatics* 2010, **11**:27.

7. Zhong Y, Wan Y-W, Pang K, Chow LM, Liu Z: **Digital sorting of complex tissues for cell type-specific gene expression profiles**. *BMC Bioinformatics* 2013, **14**:89.

8. Gaujoux R, Seoighe C: **Semi-supervised Nonnegative Matrix Factorization for gene expression deconvolution: A case study**. *Infect Genet Evol* 2012, **12**:913–921.
