# Supplementary material for: CellMapper: rapid and accurate inference of gene expression in difficult-to-isolate cell types
Source: Genome Biol. 2016 Sep 29;17:201. doi: 10.1186/s13059-016-1062-5 (PMC5043525; doi:10.1186/s13059-016-1062-5)
Supplement: Additional file 12: — Table of query genes and expression datasets used for each cell type in this study. (PDF 97 kb) [file 13059_2016_1062_MOESM12_ESM.pdf]

Additional file 13: Cell Types and Query Genes

Cell Type	Query Gene	Dataset(s) Analyzed
GABAergic Neurons	*GAD1*	Allen Brain Atlas
Noradrenergic Neurons	*SLC2A2*	Allen Brain Atlas
Serotonergic Neurons	*SLC2A4*	Allen Brain Atlas
NG2 Glia	*PDGFRA*	Allen Brain Atlas
Neurons	*L1CAM*	Allen Brain Atlas
Astrocytes	*ALDH1L1*	Allen Brain Atlas
Oligodendrocytes (Myelinating)	*MOG*	Allen Brain Atlas
Microglia	*PTPRC*	Allen Brain Atlas
Adipocytes	*FABP4*	Engreitz, et al. (2010), Lukk, et al. (2010), Zheng-Bradley, et al. (2010)
B Cells	*CD19*	Engreitz, et al. (2010), Lukk, et al. (2010), Zheng-Bradley, et al. (2010)
Cardiomyocytes	*TNNI3*	Engreitz, et al. (2010), Lukk, et al. (2010), Zheng-Bradley, et al. (2010)
Chondrocytes	*ACAN*	Engreitz, et al. (2010), Lukk, et al. (2010), Zheng-Bradley, et al. (2010)
Endothelial Cells	*TEK*	Engreitz, et al. (2010), Lukk, et al. (2010), Zheng-Bradley, et al. (2010)
Erythrocytes	*EPB42*	Engreitz, et al. (2010), Lukk, et al. (2010), Zheng-Bradley, et al. (2010)
Macrophages	*CD163*	Engreitz, et al. (2010), Lukk, et al. (2010), Zheng-Bradley, et al. (2010)
NK Cells	*NCR1*	Engreitz, et al. (2010), Lukk, et al. (2010), Zheng-Bradley, et al. (2010)
Osteoblasts	*IBSP*	Engreitz, et al. (2010), Lukk, et al. (2010), Zheng-Bradley, et al. (2010)
Platelets	*PF4*	Engreitz, et al. (2010), Lukk, et al. (2010), Zheng-Bradley, et al. (2010)
Pluripotent Stem Cells	*NANOG*	Engreitz, et al. (2010), Lukk, et al. (2010), Zheng-Bradley, et al. (2010)
Schwann Cells (Myelinating)	*MPZ*	Engreitz, et al. (2010), Lukk, et al. (2010), Zheng-Bradley, et al. (2010)
Simple Epithelial Cells	*KRT8*	Engreitz, et al. (2010), Lukk, et al. (2010), Zheng-Bradley, et al. (2010)
Skeletal Muscle Cells	*TNNT3*	Engreitz, et al. (2010), Lukk, et al. (2010), Zheng-Bradley, et al. (2010)
Smooth Muscle Cells	*MYH11*	Engreitz, et al. (2010), Lukk, et al. (2010), Zheng-Bradley,

		et al. (2010)
Stratified Epithelial Cells	*KRT5*	Engreitz, et al. (2010), Lukk, et al. (2010), Zheng-Bradley, et al. (2010)
T Cells	*CD3D*	Engreitz, et al. (2010), Lukk, et al. (2010), Zheng-Bradley, et al. (2010)
Enterocytes	*ALPI*	Intestine-specific subset of Engreitz, et al. (2010) and Lukk, et al. (2010)
Enteroendocrine Cells	*CHGA*	Intestine-specific subset of Engreitz, et al. (2010) and Lukk, et al. (2010)
Goblet Cells	*MUC2*	Intestine-specific subset of Engreitz, et al. (2010) and Lukk, et al. (2010)
Paneth Cells	*DEFA5*	Intestine-specific subset of Engreitz, et al. (2010) and Lukk, et al. (2010)
Podocytes	*PTPRO*	Kidney microarray data from Ju, et al. (2013)
