# Supplementary material for: CellMapper: rapid and accurate inference of gene expression in difficult-to-isolate cell types
Source: Genome Biol. 2016 Sep 29;17:201. doi: 10.1186/s13059-016-1062-5 (PMC5043525; doi:10.1186/s13059-016-1062-5)
Supplement: Additional file 13: — Table of positive control cell type markers selected for Figs. 2 and 3. Five markers were chosen for each of the neuron subtypes and intestinal epithelial subtypes, four for NG2 Glia, and ten for every other cell type. Genes in bold were used as negative control markers for non-target cell types in Fig. 3. (PDF 152 kb) [file 13059_2016_1062_MOESM13_ESM.pdf]

Additional file 14: Positive Control Cell Type Markers

Five positive control genes were chosen for each of the neuron subtypes and intestinal epithelial subtypes, 4 for NG2 Glia, and 10 for every other cell type. These control genes were selected without referring to our results, and whenever possible were chosen from existing lists of cell type markers

Cell Type	Positive Control Genes**	Reference(s)
GABAergic Neurons	***GAD2***, ***SLC32A1***, *SLC6A1, DLX1, DLX2*	[8]
Noradrenergic Neurons	***DBH***, ***TH***, *ADRA2A, CYB561, MAOA*	[18]
Serotonergic Neurons	***TPH2***, ***SLC6A4***, *DDC, SLC18A2, HTR1B*	[22]
NG2 Glia	***CSPG4***, *OLIG1, OLIG2, SOX10*	[23]
Neurons	***SNAP25***, ***STMN2***, *ENO2, SYN1, TUBB3*	[24]
Astrocytes	***SLC1A2***, ***SLC1A3***, *GFAP, GJB6, FGFR3, AQP4*	[24, 25]
Oligodendrocytes (Myelinating)	***PLP1***, ***MOBP***, *SOX10, MAG, MBP*	[24, 25]
Microglia	***CSF1R***, *CX3CR1, AIF1, FCGR1A, TREM2*	[26]
Adipocytes	***ADIPOQ***, ***PLIN***, *ACSL1, CEBPA, CFD, FASN, GPD1, LIPE, LPL, PPARG*	[1, 2]
B Cells	***MS4A1***, ***CD79B***, *CD79A, CD22, CD40, CR2, FCER2, IGHM, PAX5, TNFRSF13B*	[3]
Cardiomyocytes	***TNNT2***, ***MYL7***, *ACTC1, CSRP3, MYBPC3, MYH6, MYH7, MYL2, MYL4, NKX2-5*	[4]
Chondrocytes	***COMP***, ***MATN1***, *COL2A1, COL9A1, COL9A2, CHI3L1, SOX9, MATN3, CHAD, SOX6*	[5]
Endothelial Cells	***FLT1***, ***TIE1***, *CDH5, EDN1, ENG, KDR, MMRN2, PECAM1, SELE, VWF*	[6]
Erythrocytes	***GYPA***, ***RHAG***, *GYPB, GYPC, GYPE, HBA1, HBA2, HBB, RHCE, RHD*	adult hemoglobin, glycophorins, Rh blood group antigens, and erythropoietin receptor
Macrophages	***EMR1***, ***CD14***, ***CSF1R***, *ITGAM, CD68, ITGAX, CD33, FCGR1A, FCGR1B, FCGR2A*	[7]
NK Cells	***KLRD1***, ***KIR2DL1***, *KLRB1, KLRK1, KIR3DL1, NCAM1, CD244, GZMB, GNLY, PRF1*	[3]
Osteoblasts	***BGLAP***, ***SP7***, *ALPL, COL1A1, DLX5, DMP1, MEPE, OMD, RUNX2, SPP1*	[5]
Platelets	***ITGA2B***, ***GP9***, *GP1BA, ITGB3, SELP, GP5, MPL, GP6, GP1BB, TBXA2R*	[3, 5] plus *TBXA2R*
Pluripotent Stem Cells	***POU5F1***, ***ZFP42***, *SOX2, LIN28A, LIN28B, ZIC3, UTF1, FBXO15, LEFTY1, LEFTY2*	[5]
Schwann Cells (Myelinating)	***PLP1***, ***PRX***, *EGR2, MAL, MBP, NGFR, S100B, SOX10, PLP1, PMP2, PMP22*	[9, 10]

Simple Epithelial Cells	***KRT7***, ***KRT18***, *AP1M2, CDH1, CGN, DSG2, EPCAM, KRT19, OCLN, TJP3*	[11, 12] plus *AP1M2* and *EPCAM*
Skeletal Muscle Cells	***TNNI1***, ***TNNI2***, *ACTA1, CHRNA1, ENO3, MYBPH, MYF6, MYL1, MYLPF, TNNC1*	[4]
Smooth Muscle Cells	***ACTA2***, ***TAGLN***, ***DES***, *ACTG2, CNN1, CSRP1, LMOD1, LPP, SMTN, VCL*	[4]
Stratified Epithelial Cells	***KRT14***, ***KRT15***, *DSC1, DSC3, DSG1, DSG3, KRT6A, KRT16, KRT17, PKP1*	[11, 12]
T Cells	***CD3G***, ***TRA@***, *CD3E, CD4, CD8A, CD8B, CD28, CD247, TRAC, TRBC1*	TCR locus and major TCR co-receptors
Enterocytes	***FABP2***, ***SI***, *APOA1, DPP4, ANPEP*	[13, 14]
Enteroendocrine Cells	***GCG***, ***PYY***, *SST, SYP, TPH1*	[15]
Goblet Cells	***TFF3***, ***MUC5B***, *CLCA1, FCGBP, AGR2*	[16, 17]
Paneth Cells	***DEFA6***, ***REG3A***, *LYZ, REG1A, REG1B*	[19, 20]
Podocytes	*PTPRO, NPHS1, NPHS2, PODXL, WT1, MME, TJP1, TCF21, CR1*	[21]

** Genes in bold were used as negative control markers for non-target cell types in Figure 3. Negative control genes also included the set of reference genes from Eisenberg and Levanon [30]. *KLRD1* and *KIR2DL1* were not included as negative controls for T cells because they are expressed in T cells, *DES* was not included as a negative control for any muscle cell type as it is expressed in all muscle, and *EMR1* and *CD14* were not included as negative controls for microglia because they are expressed in microglia. In addition, bold genes for the neuron subtypes and intestinal epithelial lineages were only included as negative controls for other cell types in the same class (i.e. the GABAergic marker *GAD2* was used as a negative control for the other 3 neural subtypes but not any other cell type).

1. Kim JB, Spiegelman BM: **ADD1/SREBP1 promotes adipocyte differentiation and gene expression linked to fatty acid metabolism.** *Genes Dev* 1996, **10**:1096–1107.

2. Urs S, Smith C, Campbell B, Saxton AM, Taylor J, Zhang B, Snoddy J, Voy BJ, Moustaid-moussa N: **Nutrient-Gene Interactions Gene Expression Profiling in Human Preadipocytes and Adipocytes by Microarray Analysis 1 , 2**. 2004(August 2003):762–770.

3. **Cell Markers** [http://www.biolegend.com/cell_markers]

4. Nelander S, Mostad P, Lindahl P: **Prediction of cell type-specific gene modules: identification and initial characterization of a core set of smooth muscle-specific genes.** *Genome Res* 2003, **13**:1838–54.

5. **Research Topics** [http://www.rndsystems.com/]

6. Garlanda C, Dejana E: **Heterogeneity of Endothelial Cells : Specific Markers**. *Arterioscler Thromb Vasc Biol* 1997, **17**:1193–1202.

7. **CD Marker Handbook** [https://www.bdbiosciences.com/documents/cd_marker_handbook.pdf]

8. Sugino K, Hempel CM, Miller MN, Hattox AM, Shapiro P, Wu C, Huang ZJ, Nelson SB: **Molecular taxonomy of major neuronal classes in the adult mouse forebrain.** *Nat Neurosci* 2006, **9**:99–107.

9. Jessen KR, Mirsky R: **The origin and development of glial cells in peripheral nerves.** *Nat Rev Neurosci* 2005, **6**:671–82.

10. Nagarajan R, Le N, Mahoney H, Araki T, Milbrandt J: **Deciphering peripheral nerve myelination by using Schwann cell expression profiling.** *Proc Natl Acad Sci U S A* 2002, **99**:8998–9003.

11. Moll R, Divo M, Langbein L: **The human keratins: biology and pathology.** *Histochem Cell Biol* 2008, **129**:705–33.

12. Franke WW: **Discovering the molecular components of intercellular junctions--a historical view.** *Cold Spring Harb Perspect Biol* 2009, **1**:a003061.

13. Glickman RM: **Enterocyte lipid absorption and secretion**. :505–526.

14. McConnell RE, Higginbotham JN, Shifrin D a, Tabb DL, Coffey RJ, Tyska MJ: **The enterocyte microvillus is a vesicle-generating organelle.** *J Cell Biol* 2009, **185**:1285–98.

15. Gunawardene AR, Corfe BM, Staton C a: **Classification and functions of enteroendocrine cells of the lower gastrointestinal tract.** *Int J Exp Pathol* 2011, **92**:219–31.

16. Pelaseyed T, Bergstrom JH, Gustafsson JK, Ermund A, Birchenough GMH, Schutte A, van der Post S, Svensson F, Rodriguez-Pineiro AM, Nystrom EEL, Wising C, Johansson ME V, Hansson GC: **The mucus and mucins of the goblet cells and enterocytes provide the first defense line of the gastrointestinal tract and interact with the immune system**. *Immunol Rev* 2014, **260**:8–20.

17. Sheng YH, Hasnain SZ, Florin THJ, McGuckin M a: **Mucins in inflammatory bowel diseases and colorectal cancer.** *J Gastroenterol Hepatol* 2012, **27**:28–38.

18. Grimm J, Mueller A, Hefti F, Rosenthal A: **Molecular basis for catecholaminergic neuron diversity.** *Proc Natl Acad Sci U S A* 2004, **101**:13891–13896.

19. Bevins CL, Salzman NH: **Paneth cells, antimicrobial peptides and maintenance of intestinal homeostasis.** *Nat Rev Microbiol* 2011, **9**:356–68.

20. Van Beelen Granlund A, Østvik AE, Brenna Ø, Torp SH, Gustafsson BI, Sandvik AK: **REG gene expression in inflamed and healthy colon mucosa explored by in situ hybridisation.** *Cell Tissue Res* 2013, **352**:639–46.

21. Ju W, Greene CS, Eichinger F, Nair V, Hodgin JB, Bitzer M, Lee Y-S, Zhu Q, Kehata M, Li M, Jiang S, Rastaldi MP, Cohen CD, Troyanskaya OG, Kretzler M: **Defining cell-type specificity at the transcriptional level in human disease.** *Genome Res* 2013, **23**:1862–73.

22. Dougherty JD, Maloney SE, Wozniak DF, Rieger M a., Sonnenblick L, Coppola G, Mahieu NG, Zhang J, Cai J, Patti GJ, Abrahams BS, Geschwind DH, Heintz N: **The Disruption of Celf6, a Gene Identified by Translational Profiling of Serotonergic Neurons, Results in Autism-Related Behaviors**. *J Neurosci* 2013, **33**:2732–2753.

23. Dimou L, Gallo V: **NG2-glia and their functions in the central nervous system**. *Glia* 2015:n/a–n/a.

24. Zhang Y, Chen K, Sloan S a, Bennett ML, Scholze AR, O'Keeffe S, Phatnani HP, Guarnieri P, Caneda C, Ruderisch N, Deng S, Liddelow S a, Zhang C, Daneman R, Maniatis T, Barres B a, Wu JQ: **An RNA-Sequencing Transcriptome and Splicing Database of Glia, Neurons, and Vascular Cells of the Cerebral Cortex.** *J Neurosci* 2014, **34**:11929–47.

25. Cahoy JD, Emery B, Kaushal A, Foo LC, Zamanian JL, Christopherson KS, Xing Y, Lubischer JL, Krieg P a, Krupenko S a, Thompson WJ, Barres B a: **A transcriptome database for astrocytes, neurons, and oligodendrocytes: a new resource for understanding brain development and function.** *J Neurosci* 2008, **28**:264–78.

26. Elmore MRP, Najafi AR, Koike M a, Dagher NN, Spangenberg EE, Rice R a, Kitazawa M, Matusow B, Nguyen H, West BL, Green KN: **Colony-stimulating factor 1 receptor signaling is necessary for microglia viability, unmasking a microglia progenitor cell in the adult brain.** *Neuron* 2014, **82**:380–97.
